# Quadrato Motor Training (QMT) is associated with DNA methylation changes at DNA repeats: A pilot study

**DOI:** 10.1371/journal.pone.0293199

**Published:** 2023-10-25

**Authors:** Fabio Marson, Michele Zampieri, Loredana Verdone, Maria Giulia Bacalini, Francesco Ravaioli, Luca Morandi, Salvatore Gaetano Chiarella, Valerio Vetriani, Sabrina Venditti, Micaela Caserta, Antonino Raffone, Tal Dotan Ben-Soussan, Anna Reale

**Affiliations:** 1 Research Institute for Neuroscience, Education and Didactics, Fondazione Patrizio Paoletti, Assisi, Italy; 2 Neuroimaging Laboratory, Department of Physiology and Pharmacology, Sapienza University of Rome, Rome, Italy; 3 Department of Experimental Medicine, Sapienza University of Rome, Rome, Italy; 4 CNR Institute of Molecular Biology and Pathology, National Council of Research (CNR), Rome, Italy; 5 Brain Aging Laboratory, IRCCS Istituto Delle Scienze Neurologiche di Bologna, Bologna, Italy; 6 Dep. of Experimental, Diagnostic and Specialty Medicine (DIMES), University of Bologna, Bologna, Italy; 7 Dep. of Biomedical and Neuromotor Sciences, University of Bologna, Bologna, Italy; 8 Functional and Molecular Neuroimaging Unit, IRCCS Istituto Delle Scienze Neurologiche di Bologna, Bologna, Italy; 9 Institute of Sciences and Technologies of Cognition (ISTC), National Council of Research (CNR), Rome, Italy; 10 Department of Psychology, Sapienza University of Rome, Rome, Italy; 11 Dept. of Biology and biotechnologies “Charles Darwin”, Sapienza University of Rome, Rome, Italy; University of Toronto, CANADA

## Abstract

The control of non-coding repeated DNA by DNA methylation plays an important role in genomic stability, contributing to health and healthy aging. Mind-body practices can elicit psychophysical wellbeing via epigenetic mechanisms, including DNA methylation. However, in this context the effects of movement meditations have rarely been examined. Consequently, the current study investigates the effects of a specifically structured movement meditation, called the Quadrato Motor Training (QMT) on psychophysical wellbeing and on the methylation level of repeated sequences. An 8-week daily QMT program was administered to healthy women aged 40–60 years and compared with a passive control group matched for gender and age. Psychological well-being was assessed within both groups by using self-reporting scales, including the Meaning in Life Questionnaire [MLQ] and Psychological Wellbeing Scale [PWB]). DNA methylation profiles of repeated sequences (ribosomal DNA, LINE-1 and Alu) were determined in saliva samples by deep-sequencing. In contrast to controls, the QMT group exhibited increased Search for Meaning, decreased Presence of Meaning and increased Positive Relations, suggesting that QMT may lessen the automatic patterns of thinking. In the QMT group, we also found site-specific significant methylation variations in ribosomal DNA and LINE-1 repeats, consistent with increased genome stability. Finally, the correlations found between changes in methylation and psychometric indices (MLQ and PWB) suggest that the observed epigenetic and psychological changes are interrelated. Collectively, the current results indicate that QMT may improve psychophysical health trajectories by influencing the DNA methylation of specific repetitive sequences.

## Introduction

DNA methylation (DNAm), the addition of a methyl group in the carbon 5 of cytosines present in cytosine-guanine (CG) dinucleotides, is an epigenetic modification that enables heterochromatin formation and gene silencing. The DNAm pattern is established throughout prenatal developmental and differentiation processes. The subsequent maintenance of a proper methylation profile is essential for the normal functioning of the adult organism [1]. This is particularly important with regard to the repetitive elements of DNA (REs), which make up a large portion, about one-third, of the human genome [2]. The REs include tandemly repeated sequences (satellites and repeats of ribosomal DNA, rDNA) and interspersed sequences such as long interspersed nuclear elements (LINEs) and Alu sequences (Alus). Most REs are highly methylated to maintain a heterochromatic and repressed state, which is essential to preserve genome stability and control gene expression. Hypomethylation of REs is, in fact, related to chromatin relaxation, alterations in recombination processes, and unscheduled transcription. This can result in genetic alterations, which include chromosome fragility, gross chromosomal rearrangements, and insertional mutagenesis due to the mobility of REs that have retrotransposon activity, such as LINEs and Alus [3]. Moreover, hypomethylation of REs has been observed in various human diseases, from cancer to psychiatric disorders, highlighting the importance of REs methylation status [2, 4–11].

DNAm profiles can be impaired during lifetime, impacting on individual health and ageing pathways [12, 13]. Age-associated DNAm changes have been widely described [14–16] and include hypo/hyper-methylation events at specific CG sites, with a trend towards global hypomethylation of the genome due to the loss of DNAm at REs, generally associated with cellular aging and genome instability.

There is growing evidence that various environmental stressors, which include psychological stress, can cause long-lasting alterations in the methylation pattern. Such epigenetic effects may mediate the embodiment of stressful events and increase the risk of disease [13, 17–19]. In this context, hypomethylation of REs has been correlated with psychopathologies in patients with a previous history of chronic stress, childhood adversities, and emotional trauma [2, 8, 20, 21].

However, some studies suggest that the deleterious epigenetic effects induced by stress may be counterbalanced by stress management practices through remodeling of the epigenome (reviewed in [22, 23]). Meditation, a set of techniques based on control of attention and emotions, has the capability of significantly reducing stress, in addition to eliciting other beneficial emotional and cognitive effects [24–30]. Moreover, studies have demonstrated the relationship between meditation and improved psychophysical wellbeing [26, 31], as measured by instruments such as the Psychological Wellbeing scales (PWB; [32]) and the Five Facet Mindfulness Questionnaire (FFMQ; [33]).

In the context of promoting wellbeing, it is relevant to highlight the mind-body component of meditation and mindfulness. These practices have been found to enable a more physically relaxed, mentally embodied presence in the “here and now” [34, 35]. In fact, movement meditation (*i*.*e*., a practice that combines meditation and movement) and somatic focus techniques allow and enhance the top-down alpha rhythm modulation, which, in turn, sensitizes the practitioner to better detect and self-regulate sensory attention deployment. This would facilitate refocusing of attentional spotlight when the mind wanders from its somatic focus [36]. Consequently, enhanced regulation of somatic mind-wandering may be an important early stage of mindfulness training that leads to expand cognitive functions and metacognition, thereby improving psychophysical wellbeing. In addition, practices aiming to improve cognition, must not only recruit cognitive resources but challenge them continually, rendering the importance of examining the effects of movement meditations particularly relevant [37].

A recent specifically structured movement meditation that has produced interesting psychological, neurophysiological and biological effects is the Quadrato Motor Training (QMT). QMT requires whole-body coordinated movements in response to simple oral instructions within a 50 x 50 cm square, eliciting a state of divided attention between the motor response and cognitive processing (to produce the correct direction of movement according to the instruction), as well as a rapid monitoring function (reviewed in [38, 39]). In terms of its impact, QMT has been found to enhance physical, emotional, and cognitive wellbeing [38, 40, 41].

Some studies have shown links between meditation and the DNAm pattern of genes related to stress [42], immune response/inflammation [43–45], common human diseases [46] as well as genomic sites involved in epigenetic aging [47]. This evidence supports the idea that meditation-based interventions can positively influence gene expression profiles through an epigenetic “resetting” involving DNAm (reviewed in [22, 23]). However, the specific epigenetic effects of QMT have yet to be investigated.

In the present study, we aimed to evaluate possible DNAm changes resulting from QMT, with a specific focus on REs. In particular, the current study has three primary aims: (1) to examine the effects of 8 weeks of daily QMT on participants’ psychophysical wellbeing *versus* a passive control group; (2) to investigate the effects of QMT on the methylation of REs (rDNA, LINE-1, and Alus) in saliva samples; and (3) to test for possible correlations between changes of these measures.

## Materials and methods

### Enrollment of participants and experimental design

A total of 30 healthy female participants were enrolled in the study (mean age = 43.65 years; s.d. = 9.84). Written informed consent was obtained, in accordance with the Declaration of Helsinki, including informing participants of the option to withdraw at any time for any reason. This study was approved by the Department of Psychology Research Ethics and Bioethics Advisory Committee, Sapienza University of Rome, Prot. n. 0001799 October 3^rd^, 2019. Participants were semi-randomly assigned to the experimental QMT group and to the passive control group according to their availability to carry out or not to carry out the training in a defined window of time, until 15 volunteers for each group were enrolled. Participants assigned to the QMT group underwent daily QMT practice for 8 consecutive weeks. The duration of the intervention was chosen on the basis of the literature adopting meditative and mindfulness training programs [48–50]. The duration of 8 weeks has in fact proved to be adequate to provide observable and reliable effects [51]. Thus, we wanted to design the experiment in a way that it would allow comparison with results from other studies which have similar practice duration.

Collection of psychometric data and saliva samples for DNA methylation profiling was carried out on all participants at baseline (Time 1 or T1) and again after 8 weeks (Time 2 or T2). The two groups were comparable in terms of days between T1 and T2 data collection and were tested in the same period of time in order to avoid possible season-related changes in molecular data.

After collection of saliva samples, participants were seated in a quiet room to complete the psychometric tests. Six questionnaires were administered to address psychological state through psychometric evaluation. The questionnaires chosen were the Meaning in Life Questionnaire [52], Psychological Wellbeing Scales [32], Five Facet Mindfulness Questionnaire [33], Pain Catastrophizing Scale [53], Perceived Stress Scale [54], and Positive and Negative Affect Schedule [55].

The DNA methylation level of rDNA, LINE-1 and Alus repeats was determined at single nucleotide resolution on DNA isolated from saliva samples.

Several participants were excluded. The exclusion criteria related to adherence to practice were selected to have a homogeneous sample. We decided to exclude the participants who practiced for less than one standard deviation from the mean of the whole sample. The average of the days of practice was 47.93 days, and the standard deviation was 11.82. Thus, two participants were outside the range of acceptability of 36.11 days (Subject 03 = 16 days; Subject 05 = 31 days).

Two participants from both groups were excluded from the analysis because they had too many missing molecular or psychometric data (i.e., showing less than 75% of data). Thus, the final control group was comprised of 13 participants and the QMT group by 11 participants. Participants characteristics including age, menopausal status, and lifestyle habits (smoking, alcohol consumption, sports activity and meditation), are shown in Table 1 (Table 1 in S1 Appendix).

**Fig 1:**
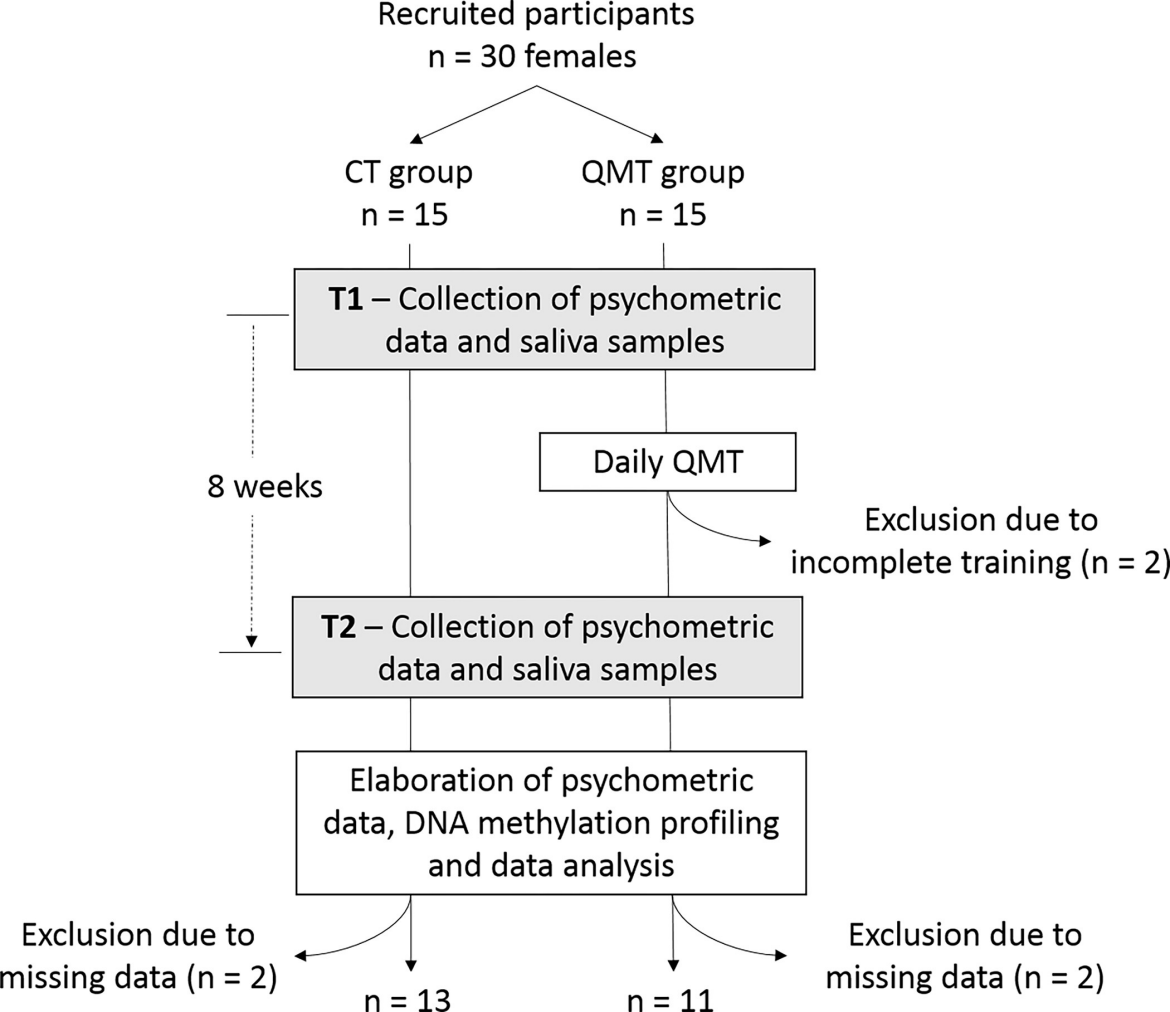
Experimental design. The figure displays the study design with the main stages of the experimental process, the sample size for each, their order and the corresponding timeline.

The research design is depicted in Fig 1.

### QMT training

QMT is a structured meditative movement training that involves sequences of vertical, horizontal, and diagonal movements performed on a 50 x 50 cm square [38, 56]. The execution of movements is guided by verbal commands and is designed to engage executive functions (e.g., divided attention and inhibition of automatic movement). After being instructed on how to perform QMT at T1, participants performed the QMT daily between T1 and T2. They were instructed to practice it preferably always in the same place (i.e., in a dedicated space in their house with enough space to freely move in front of a white empty wall) and at the same time of the day.

### Psychometric evaluation

As our objective was to address whether QMT practice could have impacts on variables associated with wellbeing, with a particular regard for stress-related behaviors and affective states, we administered several psychometric scales aimed at exploring psychological wellbeing, affective states and stress-related experiences widely used in studies adopting mindfulness and meditation training [57–60].

#### Meaning in Life Questionnaire

The Meaning in Life Questionnaire (MLQ) [52] is a 10-item questionnaire divided in two 5-item subscales: Presence of Meaning (MLQ-P) and Search for Meaning (MLQ-S). The MLQ-P subscale reflects the subjective sense that life has a clear subjective meaning and purpose. The MLQ-S subscale represents the attitude toward the active search for meaning and reflects motivation to find or increase one’s own understanding of life meaning.

#### Psychological Wellbeing Scales

The Psychological Wellbeing (PWB) questionnaire [32] is designed to assess six different core dimensions of personal psychological wellbeing: Autonomy (AU) indicating self-determination and independence from social pressure in decision making; Environmental Mastery (EM) indicating ability to manage and control complex activities involving one’s own context and environment; Positive Relations With Others (PR) indicating goodness and satisfaction related to social relations and empathic behavior; Personal Growth (PG) indicating openness to novel experiences and self-improvement; Purpose in Life (PL) indicating directedness and pursuit of defined objectives/aims; and Self-Acceptance (SA) indicating embracing different aspects of one’s self and positive attitudes about one’s past, present and future life.

#### Five Facet Mindfulness Questionnaire

The Five Facet Mindfulness Questionnaire (FFMQ) [33] is a questionnaire designed to evaluate five fundamental mindfulness-related dimensions: Observation (the ability to notice and observe different internal and external stimuli), Description (the ability to verbalize and communicate one’s thoughts and perceptions), Acting with Awareness (the ability to weigh and give volitional values to actions as opposed to absent-minded action), Non-Judgmental (the ability to refrain from judgmental evaluation of thoughts, perceptions and emotions), and Non-Reactivity (the ability to let thoughts and feelings flow without resistance). In addition, a total score is generated from the average of the five subscale scores, representing a general measure of a mindful attitude.

#### Pain Catastrophizing Scale

The Pain Catastrophizing Scale (PCS) [53] is a 13-item scale designed to evaluate and measure the subjective experience of pain and pain-related thoughts before, during or following pain experiences. In actual fact, this questionnaire does not need the respondent to be in pain at the time, but it instead explores general beliefs and thoughts about such experiences. It is divided into different subscales: Rumination describes the tendency to continuously think about painful experiences, Magnification reflects the tendency to exacerbate the subjective experience of pain, and Helplessness reflects the tendency to feel that pain cannot be avoided or reduced. A total PCS score is the general scale representing how catastrophic the subjective experience of pain can be.

#### Perceived Stress Scale

The Perceived Stress Scale (PSS) [54] is a 10-item widely used scale designed to quantify the amount of subjective feelings and thoughts about one’s own stress levels. It is a measure of the degree to which every day experiences are generally perceived and appraised as being stressful and out of control.

#### Positive and Negative Affect Schedule

The Positive and Negative Affect Schedule (PANAS) [55] is a 20-item scale measuring positive and negative affect, reflected in separate subscales. The Positive Affect (PA) subscale targets positive mood states (e.g., enthusiasm, proactivity, and joyfulness), while the Negative Affect (NA) subscale targets aversive mood states (e.g., fear, anger, and general distress). These subscales have been found sensitive to affect changes both in the short-term (state-like measurement) and over long periods such as years (trait-like measurement) [55].

### DNA methylation profiling

#### Collection of saliva, DNA extraction and sodium bisulfite treatment

Unstimulated saliva (3–4 ml) was collected from each participant at T1 and T2. Collected saliva was kept on ice, divided in aliquots into 1.5 ml tubes and centrifuged at 2500 rpm for 10’ at 4°C. The sediment, depleted of the supernatant, was stored at -80°C until DNA extraction. Genomic DNA was isolated from the sediment using the Qiamp DNA Mini Kit (Qiagen, Hilden, Germany) following the manufacturer’s protocol. The resulting DNA was quantified by Qubit dsDNA BR Assay Kit (Thermo Fisher, Waltham, MA, USA) and loaded on an agarose gel electrophoresis to check quality. Purified DNA was stored at 4°C. Genomic DNA (500 ng) was processed using the EZ DNA Methylation Kit (Zymo Research, Irvine, CA), which allows treating DNA with sodium bisulfite and converting unmethylated cytosines to uracil, leaving the methylated cytosines unmodified.

#### Library construction and target-specific deep sequencing

Targeted bisulfite sequencing is a quantitative and high throughput approach to measure DNAm of target genomic loci. Briefly, a PCR is performed on bisulfite-converted DNA using primers that target the region of interest and to which the Illumina adapter sequences are added. PCR products are indexed and subjected to next-generation sequencing. The alignment of the sequencing reads to the reference sequence allows deriving the DNAm status of each CG site in each read.

The procedures adopted were published previously [61]. Specifically, DNAm standard curves were prepared using the universal methylated and unmethylated control DNA (Millipore, Burlington, MA, USA), which were combined to generate standard samples with DNAm levels of 0, 25, 50, 75, and 100% and then subjected to bisulfite treatment. Each standard sample was sequenced in triplicate.

Bisulfite-converted DNA (5 ng) was used in PCR reactions using previously published [5, 61] target-specific primers (Table 2 in S1 Appendix). The Illumina adapter sequences TCGTCGGCAGCGTCAGATGTGTATAAGAGACAG and GTCTCGTGGGCTCGGAGATGTGTATAAGAGACAG were respectively added to each forward and reverse primer.

Sequencing libraries were generated by a two-step PCR approach. Briefly, in the first amplification step, 5 ng of bisulfite-converted DNA was amplified using 0.016 units of Phusion U Taq polymerase (ThermoFisher, Waltham, MA, United States) in 1X Phusion U buffer with the addition of 1M betaine (Merk, Darmstadt, Germany), 150 nM forward and reverse primers, 1.75 mM MgCl2 (Agena Bioscience, San Diego, CA, United States) and 200 μM dNTPs (ThermoFisher, Waltham, MA, United States) in 5 μl volume. Thermocycler conditions were set as follows: 1x cycle at 95°C for 1’ 40”; 1x cycle at 98°C for 1’; 1x cycle at 58°C for 2’; 1x cycle at 72°C for 1’; 36 x cycles at 98°C for 10”, 58°C for 40”, 72°C for 20”; 1x cycle at 72°C for 5’; maintenance at 4°C. PCR products were checked on agarose gel, pooled sample-wise and purified using the MagSi-NGS plus microspheres (MagTivio BV, Nuth, The Netherlands) as specified by the manufacturer.

In the second amplification step, 5 μl of each pooled sample was indexed using Illumina Nextera XT Index Set A, performing an 8-step PCR according to the 16S Metagenomic Sequencing Library Preparation protocol. The indexed libraries were then purified and normalized before sequencing as indicated in the Nextera Library Prep Guide. Sequencing was performed with a Micro V2 300 PE reagent kit on an Illumina MiSeq system (Illumina, San Diego, CA, USA).

#### Methylation data handling

Paired-end reads, obtained from Illumina MiSeq, were quality screened using the FastQC tool (www.bioinformatics.babraham.ac.uk/projects/fastqc/). Adapter sequences were removed using Cutadapt [62]. Subsequently, paired-end reads were merged using the PEAR tool (www.h-its.org/downloads/pear-academic/) [63], adopting a minimum number of 20 overlapping residues and a maximum read length of 450 nucleotides. The reads, assembled in FASTQ, were converted to FASTA using the Seqtk tool (https://github.com/lh3/seqtk). All handling and processing tools used were compiled in Anaconda2-based environments.

Analysis of deep bisulfite sequencing data was performed by the AmpliMethProfiler tool to estimate the DNAm level of CG sites within each target amplicon [64]. Briefly, for each sequencing target, the tool recovers all the reads with at least 80% of sequence identity (primTresh 0.8) and further filters out reads having a length differing more than 20% from that of the reference target sequence (threshLen 0.2). The Blastn tool is then used to align each read to the reference sequence, slightly modifying default parameters (dust no) in order to avoid the loss of reads with TG stretches deriving from the bisulfite-conversion of genomic regions rich in CpG sites, as previously described [65]. Binary values are assigned to each CG position in each read: 1 (methylated) is assigned when the read contains a C in the CG position, 0 (unmethylated) is assigned when the read contains a T in the CG position. Finally, the methylation ratio, calculated as the number of methylated sites out of the total number of read sites (methylated + unmethylated), across the reads was calculated for each CG position and used in further statistical analyses.

Sequencing coverage was calculated for each target region and samples with coverage < 200 were excluded from further analysis. After this filtering, the average coverage (mean ± SD) was 1965.25 ± 504.93 for LINE-1; 1300.40 ± 415.56 for Alu; 2147.26 ± 619.39 for RiboProm1; 2172.15 ± 619.39 for RiboProm2; 1817.51 ± 533.80 for 18S1; 2350.49 ± 694.14 for 18S2 and 1873.34 ± 554.18 for 28S.

### Statistical analysis

Statistical analysis was designed to explore any changes between time points among and between the groups. First, data outliers deviating by more than 2 standard deviations from the mean of each variable were identified and withdrawn from analysis. Then, each psychometric and epigenetic variable were entered into separate repeated measures ANCOVAs (rmANCOVA) with time points (T1 vs. T2) as a within-participants variable, group (QMT vs. Control) as a between-participants variable, and age as a continuous covariate. Since 12-to-37 different sites were taken in account for each gene, the p-values of the Time x Group interaction describing the differences between groups across time points were corrected for multiple comparisons adopting the FDR correction (alpha = .05, q-value = .1) with the aim to control for any Type 1 errors. In the results section, the sites showing a significant Time x Group interaction will be shown with particular emphasis on the interactions surviving the FDR correction. Observed statistical power (1-β) and partial eta squared (η_p_^2^) were computed and reported for each significant term from ANOVAs. All post-hoc comparisons were corrected for multiple comparisons adopting the Bonferroni method (computed as alpha/number of comparisons). All the p-values related to the post-hoc comparisons shown in the current manuscript are Bonferroni adjusted. To explore potential relationships between psychometric and epigenetic variables, epigenetic variables that showed a significant Time x Group interaction in the set of rmANCOVAs were further entered in a set of correlations together with the psychometric data. Since changes between baseline (T1) and follow-up (T2) were the main targets of this study, these correlations were computed on delta scores representing the difference between T2 and T1 scores. Also FDR correction with the same parameters adopted for ANOVAs was further applied to the p-values obtained from the correlations.

The data set supporting this study is provided as supplementary information (S1 Data).

All analyses were conducted on SPSS (SPSS Inc., Chicago IL, vers. 26.0).

## Results

### Psychometric indices

#### MLQ

**Fig 2:**
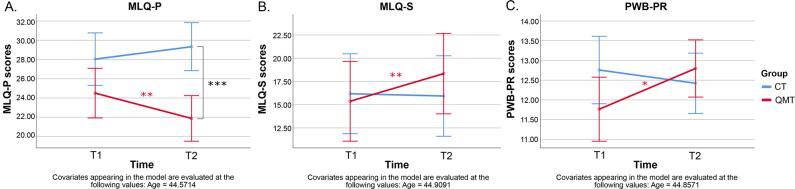
MLQ and PWB subscale scores by time and group representing Time x Group interaction results. (A) MLQ-Presence of Meaning (MLQ-P) subscale scores at Time 1 (T1) and Time 2 (T2) by group; (B) MLQ-Search for Meaning (MLQ-S) subscale scores at T1 and T2 by group; (C) PWB-Positive Relations (PWB-PR) subscale scores at T1 and T2 by groups. Data are estimated marginal mean ± 95% CI. * p < .05, ** p < .01 and *** p < .001 post-Bonferroni correction.

MLQ Presence of Meaning (MLQ-P) total scores showed a significant Time x Group interaction [F(1,18) = 9.62, p < .01, η_p_^2^ = .35, 1-β = .83] and a significant Time x Age interaction [F(1,18) = 4.5, p < .05, η_p_^2^ = .20, 1-β = .51]. Post-hoc Bonferroni correction of the Time x Group interaction revealed that the groups’ total MLQ-P scores were not statistically different at T1 (QMT T1 = 24.5, Control T1 = 28.04; p = .08), but did show a significant difference at T2 (QMT T2 = 21.8, Control T2 = 29.3, p < .001) (Fig 2A). Moreover, while the Control group showed a statistically non-significant MLQ-P score change over time (Control T1 = 28.04, Control T2 = 29.3; p = .15), the QMT group showed a significant decrease in MLQ-P scores after training compared to before training (QMT T1 = 24.5, QMT T2 = 21.8; p < .01). In addition, a significant main effect of Time [F(1,18) = 5.33, p < .05, η_p_^2^ = .03, 1-β = .59] and a significant main effect of Group [F(1,18) = 10.29, p < .01, η_p_^2^ = .36, 1-β = .86] were observed.

**Fig 3:**
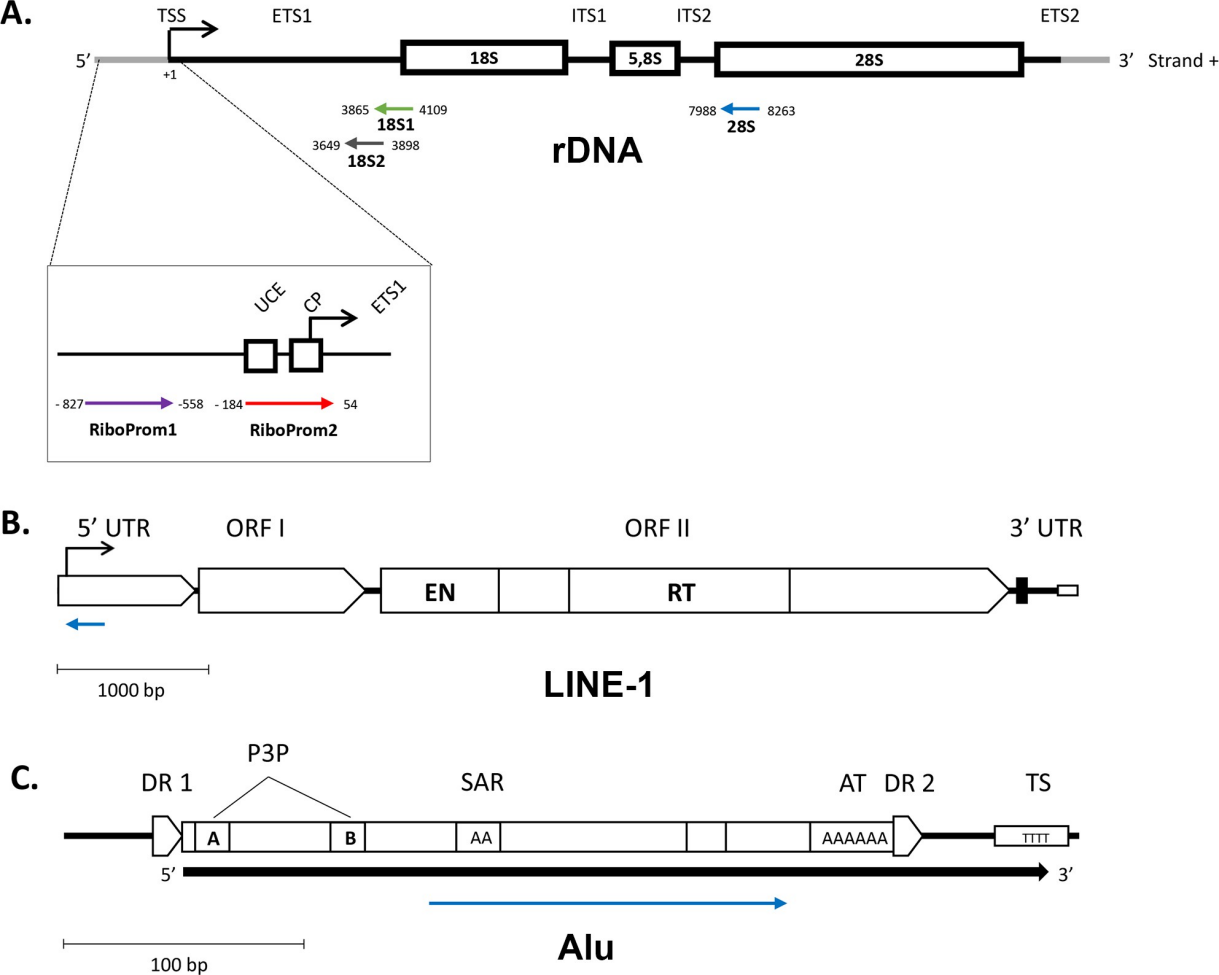
Structure of the rDNA, LINE-1 and Alu units and localization of target regions assessed. (A) General structure of the rDNA unit. The position of each amplicon (RiboProm1, violet arrow; RiboProm2, red arrow) included in the targeted-bisulfite sequencing assay is defined by its distance (in base pairs) from the transcription start site (TSS) relative to the 45S pre-ribosomal RNA transcript (chrUn_gl000220, GRCh37/hg19). UCE = upstream control element; CP = core promoter; TSS = transcription start site; ETS = external transcribed spacer; ITS = internal transcribed spacer; 18S = 18S rRNA coding region; 5.8S = 5.8S rRNA coding region; 28S = 28S rRNA coding region. (B) General structure of the LINE-1 unit [adapted from [69]]. The amplicon (blue arrow) included in the targeted-bisulfite sequencing assay is located next to the transcription start sites (black arrow), relative to the Human LINE-1 (L1.4) repetitive element DNA sequence (GenBank: L19092.1). UTR = untranslated region; ORF = open reading frame; EN = endonuclease coding region; RT = reverse transcriptase coding region. (C) General structure of the Alu unit [adapted from [70]]. The amplicon (blue arrow) included in the targeted-bisulfite sequencing is located centrally inside the repeated unit, between the RNA polymerase III promoters (P3P A and P3P B) and the long A-rich region (AT). DR = direct repeat; SAR = small A-rich region; TS = T-rich transcription terminator sequence. The black filled arrow indicates the typical Alu transcript. The orientation of the arrows representing the amplicons in (A), (B) and (C) indicates the 5’-3’ direction of the sequenced DNA molecule. Bp = base pairs.

Further analysis of the Time x Age interaction showed a significant decrease in the difference between MLQ-P scores at T1 and T2 as a function of age, suggesting that the older the participants, the lower is the MLQ-P difference over time, without regard to group (Fig 3A in S1 Appendix).

Search for Meaning (MLQ-S) subscale scores showed a significant main effect of Age [F(1,19) = 7.11, p < .05, η_p_^2^ = .27, 1-β = .71] and a significant Time x Group interaction [F(1,19) = 4.81, p < .05, η_p_^2^ = .20, 1-β = .55]. Post-hoc Bonferroni correction of the Time x Group interaction indicated that both groups’ MLQ-S scores were not statistically different either at T1 (QMT T1 = 15.36, Control T1 = 16.17; p = .79) or T2 (QMT T2 = 18.34, Control T2 = 15.93; p = .44) (Fig 2B). The main effect of Age suggests that MLQ-S scores tended to decrease with age without regard to group (Fig 3B in S1 Appendix). However, the QMT group showed a significant increase in MLQ-S scores after QMT compared to pre-training (T1 = 15.36, T2 = 18.34; p < .01), while the Controls showed no changes in MLQ-S scores over time (T1 = 16.17, T2 = 15.93; p = .80) (Fig 2B).

All other main effects and interactions were not significant (all p-values > .41).

#### PWB questionnaire

The Positive Relations With Others (PR) subscale scores of the PWB questionnaire showed a significant main effect of Age [F(1,18) = 5.15, p < .05, η_p_^2^ = .22, 1-β = .57] and a significant Time x Group interaction [F(1,18) = 5.84, p < .05, η_p_^2^ = .24, 1-β = .63]. The main effect of Age suggests that PWB-PR scores decreased with age without group differences (Fig 3C in S1 Appendix). Post-hoc Bonferroni correction of the Time x Group interaction showed that while both groups’ PWB-PR scores were not significantly different at either T1 (QMT T1 = 11.76, Control T1 = 12.75; p = .11) or T2 (QMT T2 = 12.79, Control T2 = 12.42; p = .48) (Fig 2C), a significant increase in PWB-PR scores was observed for the QMT group at T2 (T1 = 11.76, T2 = 12.79; p < .05), while the Control group showed no changes over time (T1 = 12.75, T2 = 12.42; p = .40). All other main effects and interactions were not significant (all p-values > .53).

The PWB questionnaire—Personal Growth (PG) subscale scores showed a significant main effect of Age [F(1,17) = 8.98, p < .01, η_p_^2^ = .34, 1-β = .80]. The main effect of Age indicates that PWB-PG scores decreased with age without differences between groups (Fig 3D in S1 Appendix). Moreover, the main effect of Group approached significance [F(1,17) = 4.35, p = .052, η_p_^2^ = .20, 1-β = .50] suggesting a trend towards a difference between groups, with the QMT group showing slightly higher mean scores compared to the Controls (QMT = 12.54, Control = 11.32).

All other main effects and interactions were not significant (all p-values > .41) and all other PWB subscales (i.e., Autonomy, Environmental Mastery, Purpose In Life, and Self-Acceptance) showed no significant main effects or interactions (all p-values > .07).

#### FFMQ

The FFMQ Description subscale scores showed a significant main effect of Age [F(1,18) = 8.97, p < .01, η_p_^2^ = .33, 1-β = .81], suggesting that the scores decrease with age regardless of group (Fig 3E in S1 Appendix). All other main effects or interactions of the Description subscale were not significant (all p-values > .09).

The FFMQ Act With Awareness subscale, the FFMQ Non-Judgmental subscale and the FFMQ total score showed a significant main effect of Age [F(1,19) = 5.03, p < .05, η_p_^2^ = .21, 1-β = .56; F(1,18) = 6.24, p < .05, η_p_^2^ = .25, 1-β = .65; F(1,19) = 8.81, p < .01, η_p_^2^ = .31, 1-β = .80, respectively], indicating that scores decrease with age without differences between groups (Fig 3F–3H in S1 Appendix).

All other main effects or interactions of the FFMQ Total scale were not significant. Additionally, the Observation and the Non-Reactivity subscales of the FFMQ showed no significant main effects or interactions (all p > .09).

#### PCS, PSS and PANAS

These questionnaires showed no significant main effect or interactions in their subscales (all p-values > .09).

### DNA methylation profile

#### Structure of the rDNA, LINE-1 and Alu units

The rDNA unit encodes the 45S pre-ribosomal RNA (RNA45S), which is the precursor for 18, 5.8 and 28S rRNAs. Each unit is repeated a varying number of times in the genome (30–40 rDNA repeats) at the short arm of five acrocentric chromosomes (HSA13, HSA14, HSA15, HSA21 and HSA22), resulting in approximately 400 rDNA copies in diploid cells. The promoter region was analyzed at the RiboProm1 and RiboProm2 targets (Fig 3A), as reported in previous studies [5]. RiboProm1 is upstream of the rDNA promoter, whereas RiboProm2 encompasses the upstream control element (UCE), the central promoter (CP) and the transcription start site (TSS).

The 18S and 28S gene bodies were analyzed at the 18S1, 18S2 and 28S targets, designed to cover the 5’ end of the respective sequences in the rDNA unit (Fig 3A), as described in previous studies [61, 66, 67]. The RiboProm1, RiboProm2, 18S1, 18S2 and 28S targets contain 37, 26, 27, 13 and 30 CG sites, respectively (Fig 1, upper panel, in S1 Appendix).

LINE-1 and Alu repeats are sequences dispersed throughout the genome, present in nearly all chromosomes. The human genome contains about 600,000 LINE-1 and 1,000,000 Alu repeats that contribute to about 17% and 11% of the total genomic DNA [68], respectively. Methylation of LINE-1 and Alu repeats was analyzed at target sequences described in previous studies [5]. The LINE-1 target is in the 5’ untranslated region (5’ UTR) of the repeat unit, near the transcription start site (Fig 3B). The Alu target is in the body of the repeat unit (Fig 3C). The LINE-1 and Alu targets contain 18 and 12 CG sites, respectively (Fig 2 in S1 Appendix).

#### DNA methylation level of rDNA, LINE-1 and Alus repeats

The DNA methylation level of rDNA repeats, LINE-1 and Alus was determined at the single CG dinucleotide level. Position of CG sites within DNA targets and the corresponding methylation level found in the Control and QMT groups at experimental times T1 and T2 are given in Table 3 (left side) and plotted in Figs 1 and 2 in S1 Appendix. To trace QMT-associated methylation changes, data were compared between groups (QMT, Control) and experimental times (T1, T2) by a Time x Group age-adjusted rmANCOVA (Table 3, right side, in S1 Appendix).

The methylation level of seven CG sites of the rDNA unit showed significant Time x Group interactions. Three CG sites belonged to RiboProm regions: RiboProm1 CG 210 [F(1,18) = 6.87, p < .05, η_p_^2^ = .27, 1-β = .69] and RiboProm2 CG 56 [F(1,19) = 5.03, p < .05, η_p_^2^ = .21, 1-β = .56] and CG 204 [F(1,21) = 5.46, p < .05, η_p_^2^ = .21, 1-β = .61]. Four sites belonged to the 18S and 28S coding regions: 18S1 CG 75 [F(1,20) = 5.03, p < .05, η_p_^2^ = .20, 1-β = .57], CG 120 [F(1,19) = 6.94, p < .05, η_p_^2^ = .27, 1-β = .70], CG 179 [F(1,19) = 9.27, p < .01, η_p_^2^ = .33, 1-β = .82] and 28S CG 246 [F(1,19) = 7.19, p < .05, η_p_^2^ = .27, 1-β = .72]. Other rDNA CG sites were non-significant for Time x Group interactions (all p-values > .065).

Three CG sites belonging to the interspersed DNA repeats showed a significant Time x Group interaction, specifically Alu CG 94 [F(1,19) = 5.37, p < .05, η_p_^2^ = .22, 1-β = .60], LINE-1 CG 99 [F(1,20) = 8.11, p < .01, η_p_^2^ = .29, 1-β = .77] and CG 173 [F(1,19) = 10.70, p < .01, η_p_^2^ = .36, 1-β = .87]. Other interspersed DNA repeats sites were non-significant (all p-values > .08).

Three interactions survived the FDR correction: 18S1 CG 179 and LINE-1 CG 99 and CG 173 (for a complete overview of descriptive statistics and results see Table 3 in S1 Appendix).

**Fig 4:**
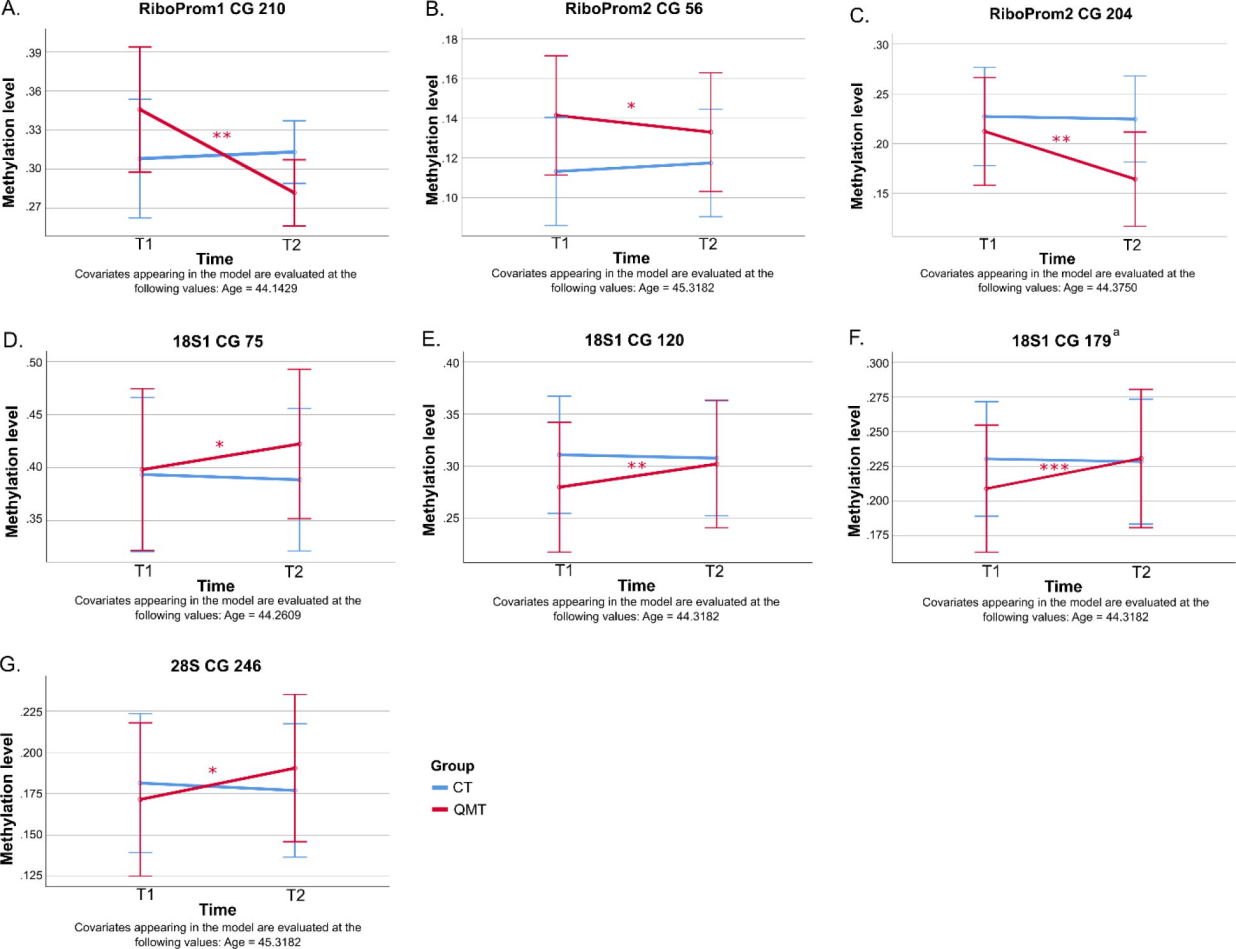
Methylation levels by Time (T1, T2) and Group (QMT, Control) representing Time x Group interaction results. Methylation levels for QMT and Control (CT) groups over time for: (A) RiboProm1 CG 120, (B) RiboProm2 CG 56, (C) RiboProm2 CG 204, (D) 18S1 CG 75, (E) 18S1 CG 120, (F) 18S1 CG 179, (G) 18S1 CG 246. Data are estimated marginal mean ± 95% CI. * p < .05, ** p < .01 and *** p < .001 post-Bonferroni correction. ^a^Time x Group interaction that survived the FDR correction (q = .1).

Age did not have a significant effect on the above associations, except for RiboProm1 CG 210 which showed a significant main effect of Age [F(1,18) = 5.38, p < .05, η_p_^2^ = .23, 1-β = .59]. In fact, although overall methylation levels at this site tend to decrease with age, no significant mean methylation differences were noted between groups (Fig 4 in S1 Appendix).

Pairwise comparison analysis was performed for all Time x Group interactions using the post-hoc Bonferroni correction. For the rDNA unit, all CG sites showed significant changes in methylation levels across time (T1 to T2) in the QMT group. However, while methylation levels decreased at promoter sites (RiboProm1 CG 210; RiboProm2 CG 56 and CG 204) (Fig 4A–4C), they increased at gene body sites (18S1 CG 75, CG 120 and CG 179; 28S CG 246) (Fig 4D–4G). In contrast, the Control group showed no significant changes from T1 to T2, nor were they statistically different from the QMT group at either time points.

**Fig 5:**
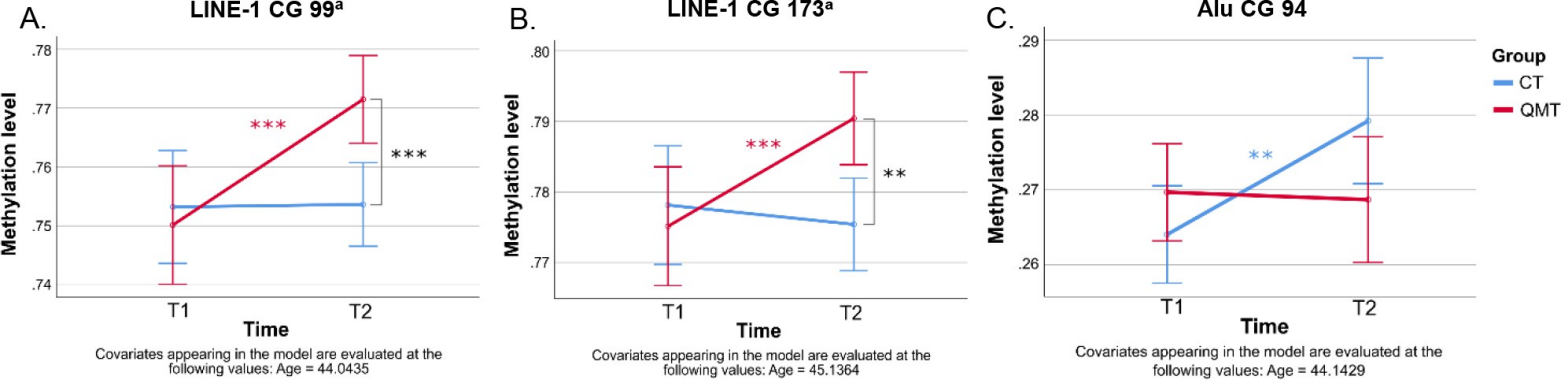
Methylation levels by Time (T1, T2) and Group (QMT, Control) representing Time x Group interaction results. Methylation levels for QMT and Control (CT) groups over time for (A) LINE-1 CG 99, (B) LINE-1 CG 173 and the (C) Alu CG 94. Data are estimated marginal mean ± 95% CI. ** p < .01 and *** p < .001 post-Bonferroni correction. ^a^Time x Group interaction that survived the FDR correction (q = .1).

For interspersed DNA repeats, LINE-1 CG 99 and CG 173 showed increased methylation levels for the QMT group from T1 to T2, whereas the Control group showed no significant change. While methylation levels were significantly higher in QMT compared to the Control group at T2, no group difference was evident at T1 (Fig 5A and 5B). Conversely, Alu CG 94 showed a significant methylation increase across time (T1 to T2) for the Control group, whereas no significant changes were found for the QMT group, or between Control and QMT groups at either time points (Fig 5C).

### Correlation between psychometric variables and CG methylation levels

Associations between changes over time in psychometric and methylation indices (Δ, T2-T1 difference) were assessed by means of a bivariate linear correlation analysis (Table 4 in S1 Appendix). Analyses examined only indices showing significant Time x Group interaction in the previously described set of rmANCOVAs.

The changes in MLQ’s Presence of Meaning subscale (MLQ-P) scores negatively correlated with changes in methylation levels of 18S1 CG 75 (r = -.642, p < .01), CG 120 (r = -.531, p < .05) and CG 179 (r = -.599, p < .01), and 28S CG 246 (r = -.533, p < .05). The changes in MLQ’s Search for Meaning subscale (MLQ-S) scores correlated with changes in methylation levels of 18S1 CG 120 (r = .481, p < .05) and CG 179 (r = .451, p < .05). The changes in Psychological Wellbeing Scales’ Positive Relations With Others subscale (PWB-PR) scores correlated with changes in methylation levels of 18S1 CG 120 (r = .572, p < .05) and LINE-1 CG 173 (r = .620, p < .01). However, the association of 18S1 CG 120 and 28S CG 246 with MLQ-P subscale score, together with the association of 18S1 CG 120 and CG 179 with MLQ-S subscale score, did not survive the FDR correction (for a complete report of all correlations results, see Table 4 and Fig 5 in S1 Appendix).

## Discussion

The current study shows that eight weeks of daily QMT in healthy women resulted in some psychometric and methylation changes compared to a passive control group. In particular, the QMT intervention was correlated with modified psychological indices in association with DNAm profile changes at various repetitive DNA sequences.

### Changes at the level of psychometric measurements

In contrast to the Control group’s lack of change in psychometric indices over time, changes were observed in the QMT group after eight weeks of QMT practice (T2) compared to baseline (T1). Interestingly, while Presence of Meaning (MLQ-P) was reduced following QMT, Search for Meaning (MLQ-S) was increased. One possible interpretation of this finding may relate to the fact that QMT was found to diminish the automaticity of habitual thinking patterns by improving motor and cognitive inhibition [38], reflectivity [56] and creativity [71, 72]. Consequently, the daily engagement in the highly mindful QMT practice could induce the observed changes in MLQ-P and MLQ-P scores by shifting a more habitual automatic daily behavior into a more dynamic pro-active attitude, thus stimulating further search for new meaning.

QMT practice was also associated with increased scores in the Positive Relations With Others subscale of the PWB scales. The effect of QMT on social perception may stem from the impacted brain areas. In fact, previous studies have shown QMT can improve affective balance [73], as well as increase synchronization between bilateral limbic and prefrontal areas [74]. This may promote functional integration of affective and non-affective executive functions [39], suggesting that QMT can influence complex affective processes by promoting neural plasticity in emotion-related brain areas thereby affecting the quality and subjective appraisal of social interactions. Further studies are needed to clarify the nature, magnitude, and neurocognitive mechanisms underlying this change.

Age was found to covariate with scores on the Personal Growth subscale of the PWB scales, as well as the FFMQ total score and three of its subscales (*i*.*e*., Description, Acting With Awareness, Non-Judgmental), indicating that lower scores were a function of greater age. It is possible that the PWB construct of personal growth (openness to new experiences and self-improvement) evoked the inter-relation with age, as this type of goal seeking and positive attitude towards the future is often observed more in youth and tends to decrease with age by becoming less relevant [75]. Concerning the FFMQ, a previous study found a positive relationship between age and the Acting With Awareness FFMQ subscale scores [31]. However, other authors debated the inter-relation of FFMQ scores with age showing that meditation history correlated with age while FFMQ total score did not [76], suggesting that different variables are involved in these relationships.

### Changes at the DNAm level

DNAm analyses were performed on saliva, one of the most easily accessible sample types and a good proxy for DNAm changes in other tissues such as brain and blood [77, 78]. The majority of methylation changes that varied significantly over time were associated with QMT training. Significant DNAm changes were found associated with QMT practice in specific CG sites located at REs such as rDNA and interspersed LINE-1. These findings suggest QMT practice might contribute to modulating DNAm profiles at the level of specific REs, which could result in positive effects such as increased genome stability.

The observed changes appear to be target-specific, suggesting that the effect of QMT practice depends on the functional role of the REs. For instance, at the rDNA targets, promoter regions underwent hypomethylation, while 18S and 28S gene body regions underwent hypermethylation. Both processes are of potential functional relevance because they stimulate rRNA transcription [79, 80], which in turn favors ribosome biogenesis and protein synthesis. In contrast, loss of this bimodal methylation profile has been associated with cellular senescence [81, 82] and high suicide rates among individuals abused as children [83]. Moreover, it has been reported that methylation of both promoter and coding regions increases during aging leading to an age-related decline in rDNA levels [61, 84, 85]. Therefore, QMT training may represent an effective tool to promote an epigenetic retuning of rRNA synthesis via counteracting DNAm alteration caused by aging.

The effect of QMT appears to be target-specific even for interspersed transposable elements, as it affects LINE-1, rather than Alu sequences. This probably reflects differences in the biological and functional characteristics of these repetitive elements, but still remains unclear [86]. Nevertheless, the association between QMT practice and the methylation level of LINE-1 is particularly significant since it able to differentiate the QMT group from the Controls group at T2. The hypermethylated state of these regions plays extremely important structural and regulatory roles in the entire genome. In fact, hypermethylation of LINE-1 ensures the genetic stability of the genome by counteracting the retrotransposition process and controls the global 3D structure of chromatin [87]. Conversely, failure of these regulatory functions due to loss of LINE-1 methylation is associated with diseases, primarily cancer [88–90]. Loss of methylation is also observed in healthy people during the aging process [91–94], as well as in age-related diseases and conditions such as menopause [95], frailty [96] and Alzheimer’s disease [97, 98]. Significantly, recent evidence suggests that transcriptional derepression of LINE-1 sequences during cellular senescence activates a type-I interferon (IFN-I) response and sustains age-associated chronic inflammation (inflammaging) [4]. Accordingly, our finding of increased LINE-1 methylation levels associated with QMT provides preliminary evidence that consistent practice of this movement meditation may induce favorable health outcomes.

Although statistically significant and functionally relevant, the vast majority of both hypomethylated and hypermethylated CG sites associated with QMT exhibited quite subtle changes. The small magnitude of mean differences across time points, the small sample size, and the naturally high variability of DNA repeats methylation among the general population [99], might explain the suboptimal statistical power (1-β frequently below .8) and the classification of many discovered associations as false positive findings after the FDR correction. This is plausible and probably accounts for the increase in Alu CG 94 methylation across time points in the control group.

The correlation between the CG methylation levels of 18S, 28S, and LINE-1 regions and scores of the MLQ and PWB questionnaires indicates that the observed epigenetic and psychological changes are inter-related and that better wellbeing is concomitant with favorable epigenetic remodeling. This possibility is supported by previous studies. In particular, the association of 18S and 28S rDNA methylation levels with indices of Presence of Meaning in Life, Searching for Meaning, and Positive Relations With Others reported here, is congruent with previous findings linking the loss of rDNA methylation with states of psychological distress/pathology [100, 101]. Similarly, the positive association found between LINE-1 methylation and the Positive Relations With Others subscale is consistent with the hypothesis that LINE-1 hypomethylation is associated with unhealthy lifestyles [102] and mental disorders such as schizophrenia [8] and posttraumatic stress disorder [9].

### Strengths and limitations

The main outcome of the current study is the evidence that an important epigenetic mechanism like DNAm variations at the genomic DNA repeats may contribute to explain the physiological and psychological changes induced by the QMT practice. To our knowledge, this is the first study to describe an effect of meditation on DNAm of repeated elements.

Several study limitations must be acknowledged, beginning with the small, homogeneous (age and gender) cohort, which may have affected the internal validity of inferences and the degree to which the results can be generalized. Notably, although the effect sizes (η_p_^2^) were above .14 for most of the significant associations, indicating a good explanatory power of the Time x Group interaction, the statistical power (1-β) was generally below the commonly accepted minimum threshold of .8. This result, together with the outcome of the FDR correction, suggests that our data have good explanatory power but some improvements will be necessary to guarantee a better protection against Type II errors. The small sample size also precluded multivariable and sub-group analyses, as well as investigation of other potential confounding variables beyond age. Finally, given the tissue-specific nature of DNAm and the cellular heterogeneity of saliva [103], changes in cell type composition of saliva are additional potential sources of variation that may have impacted both case-control and pre-post analyses.

Despite these limitations, the results show biological plausibility and can act as a basis for achieving in the future a more complete picture of the effect of meditation practices on DNA methylation profile.

## Conclusions

Considering the relative long-term stability of DNAm profiles, our results suggest that QMT may improve psychological health trajectories by influencing, at least in part, the DNAm status of specific repetitive sequences. Larger cohort studies with longer observation durations are needed to confirm these noteworthy preliminary findings.

## Supporting information

S1 Appendix (DOCX)

S1 Data Minimal anonymized dataset. (XLSX)
