# Supplementary material for: Quadrato Motor Training (QMT) is associated with DNA methylation changes at DNA repeats: A pilot study
Source: PLoS One. 2023 Oct 25;18(10):e0293199. doi: 10.1371/journal.pone.0293199 (PMC10599555; doi:10.1371/journal.pone.0293199)
Supplement: S1 Appendix — (DOCX) [file pone.0293199.s001.docx]

**S1 appendix**

**
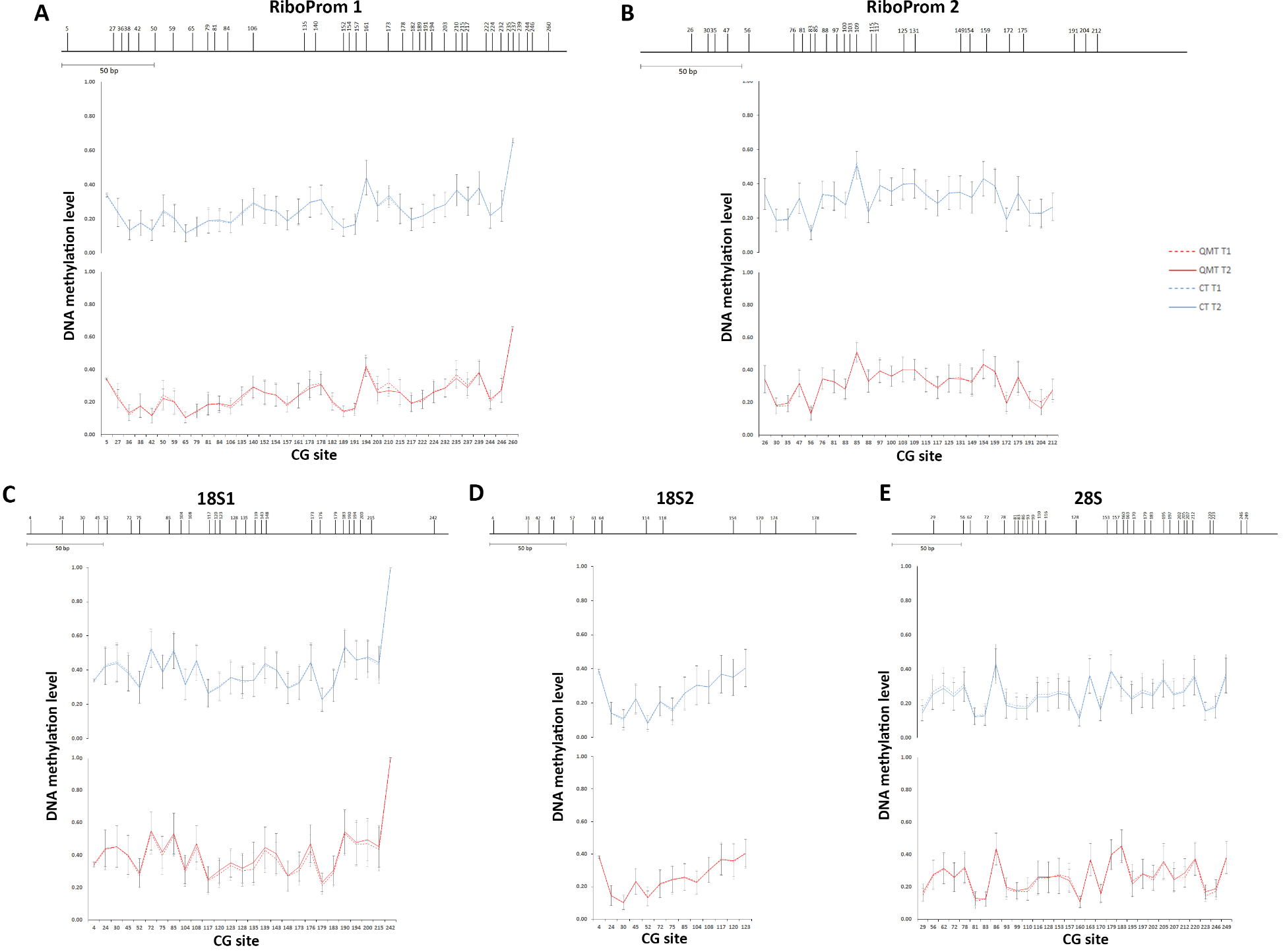
**

**Fig 1. Relative position and methylation level of CG sites of rDNA targets**. Position and methylation level of CG sites measured in QMT and Control groups at T1 and T2 times for the DNA targets RiboProm1 (A), RiboProm2 (B), 18S1 (C), 18S2 (D) and 28S (E). Each CG site is identified by a number indicating the nucleotide position relative to the 5' end of the sequenced DNA molecule. Methylation level is given as mean ± SD. Bp = base pairs.


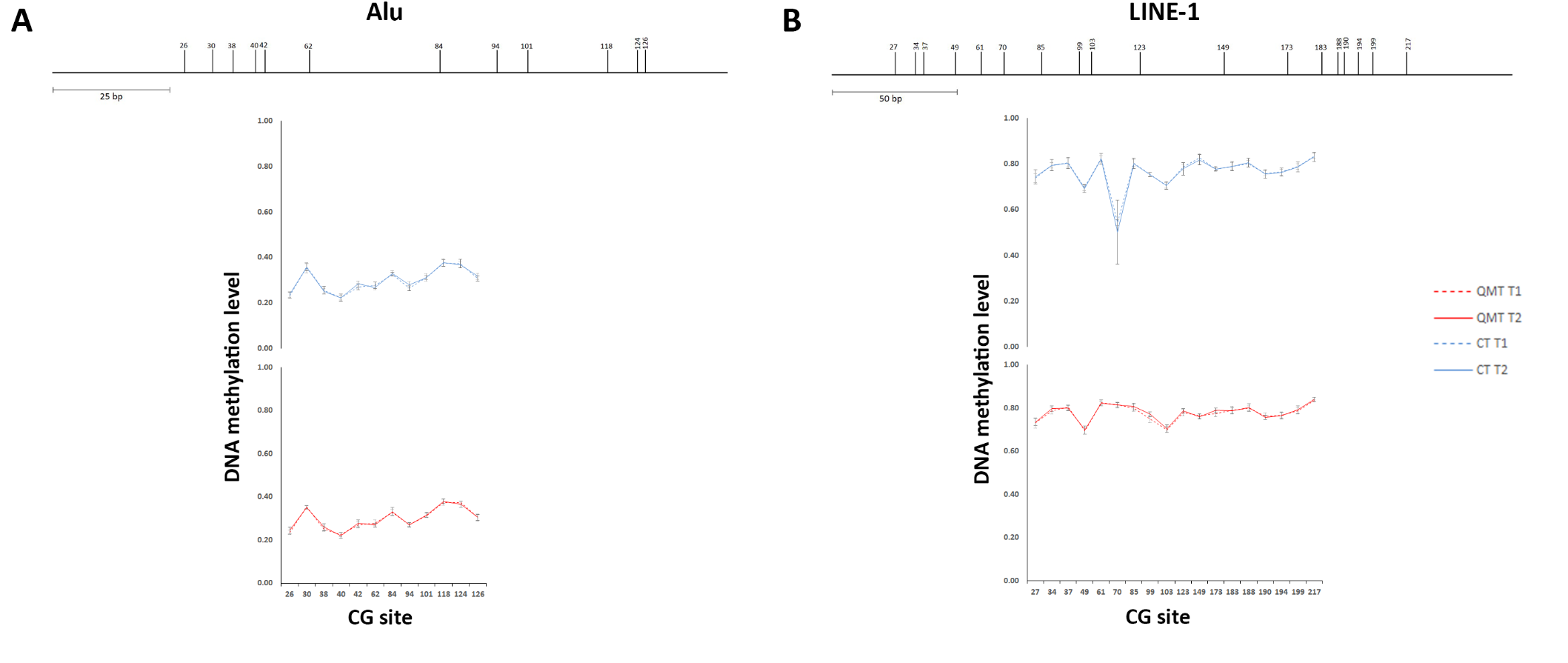


**Fig 2.** **Relative position and methylation level of CG sites of Alu and LINE-1 targets.**

Position and methylation level of CG sites measured in QMT and Control groups at T1 and T2 times for the Alu (A) and LINE-1 (B) DNA targets. Each CG site is identified by a number indicating the nucleotide position relative to the 5' end of the sequenced DNA molecule. Methylation level is given as mean ± SD. Bp = base pairs.


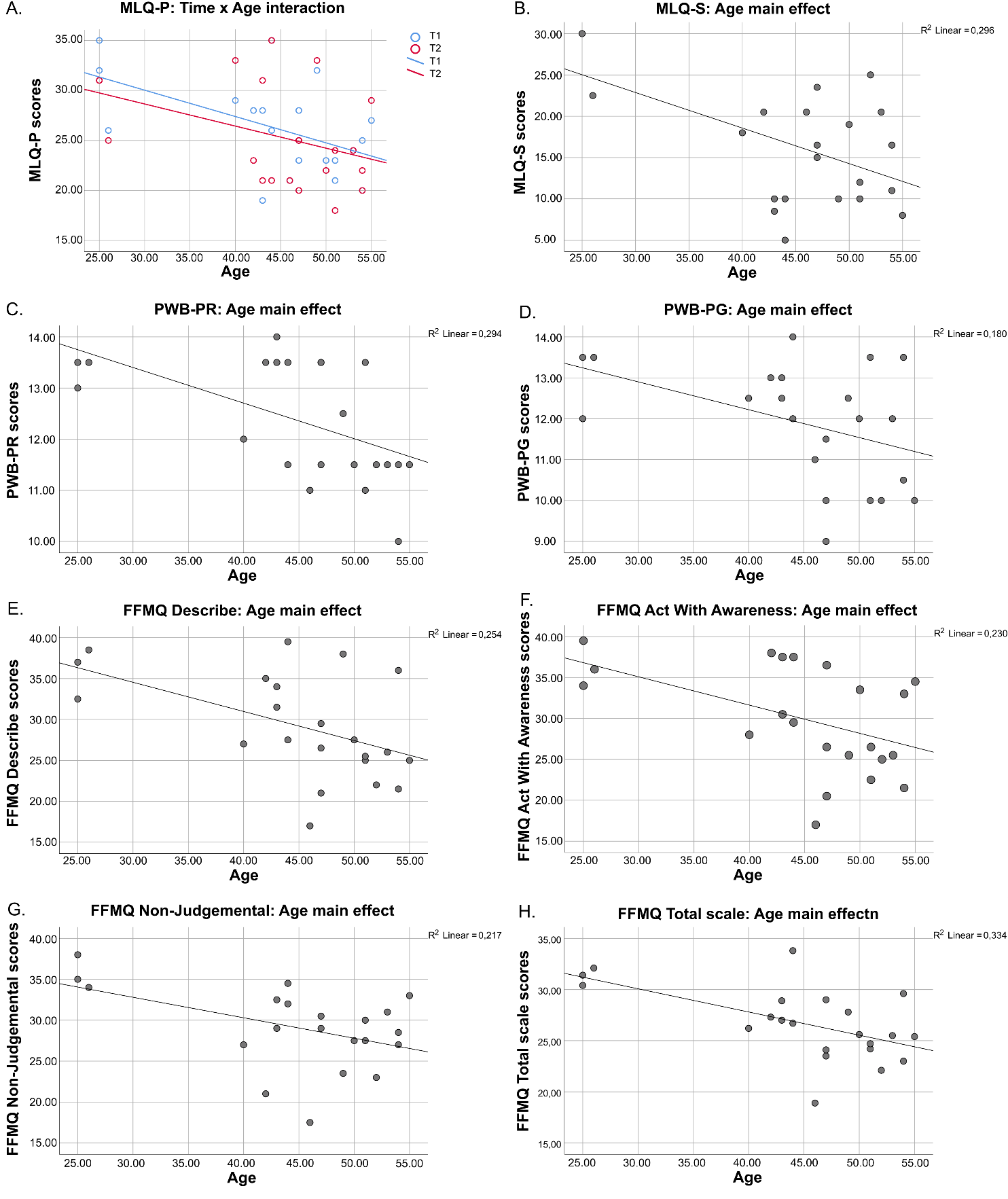


**Fig 3. Scatterplots representing results related to main effect of Age or interaction between Time and Age of rmANCOVA for psychometric indices.**

In detail, scores of Presence of Meaning In Life (MLQ-P) by Time (T1, T2) and Age (3A), Scores of Searching for Meaning in Life (MLQ-S; 3B), Psychological Wellbeing – Positive Relations (PWB-PR; 3C), Psychological Wellbeing – Personal Growth (PWB-PG; 3D), Five Facet Mindfulness Questionnaire (FFMQ) – Description (3E), Act With Awareness (3F), Non-Judgmental (3G), and the Total Score of FFMQ (3H) by Age.


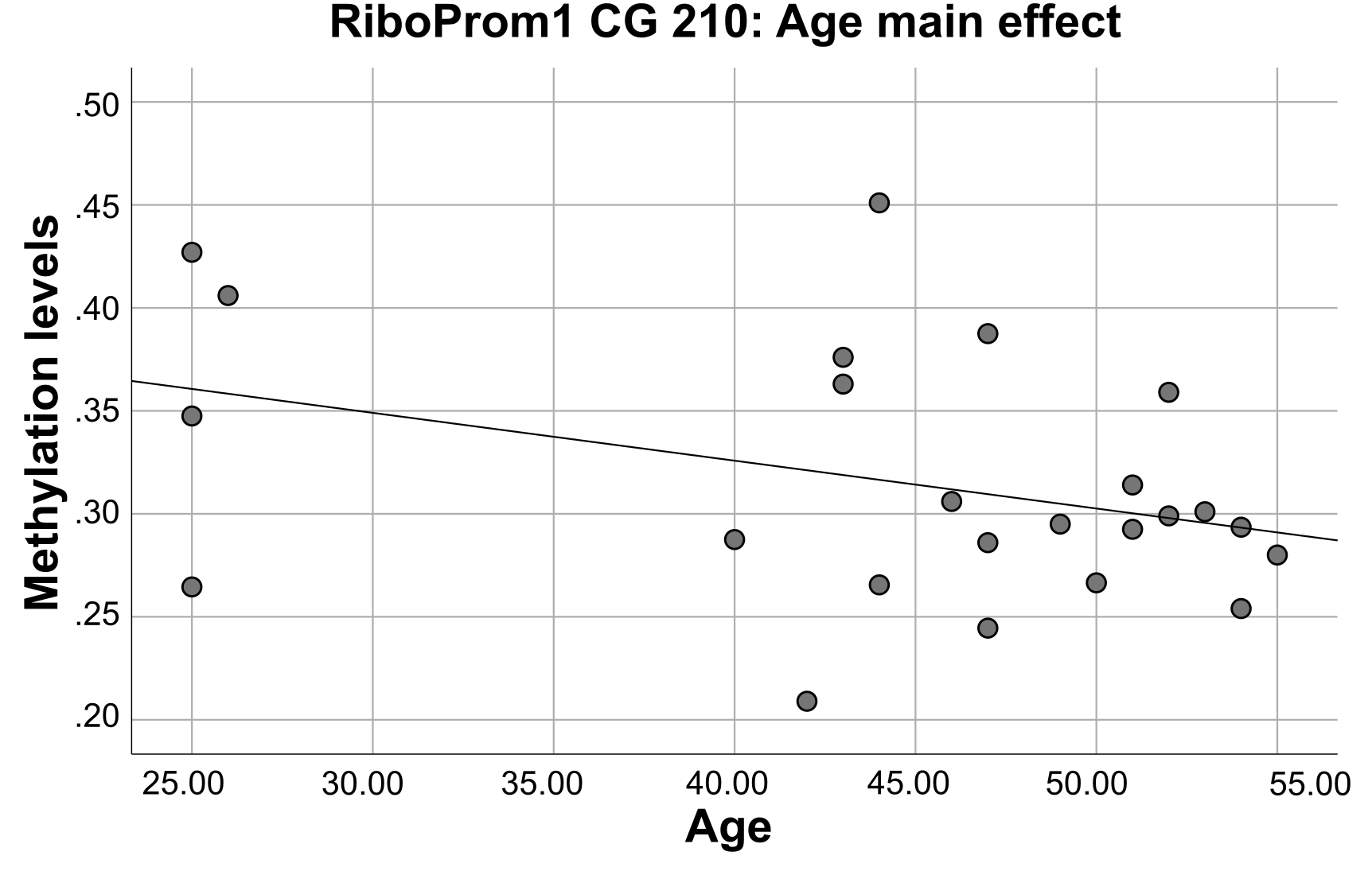


**Fig 4.** **Scatterplots representing the main effect of Age in the methylation levels of the RiboProm1 CG 210 site.**


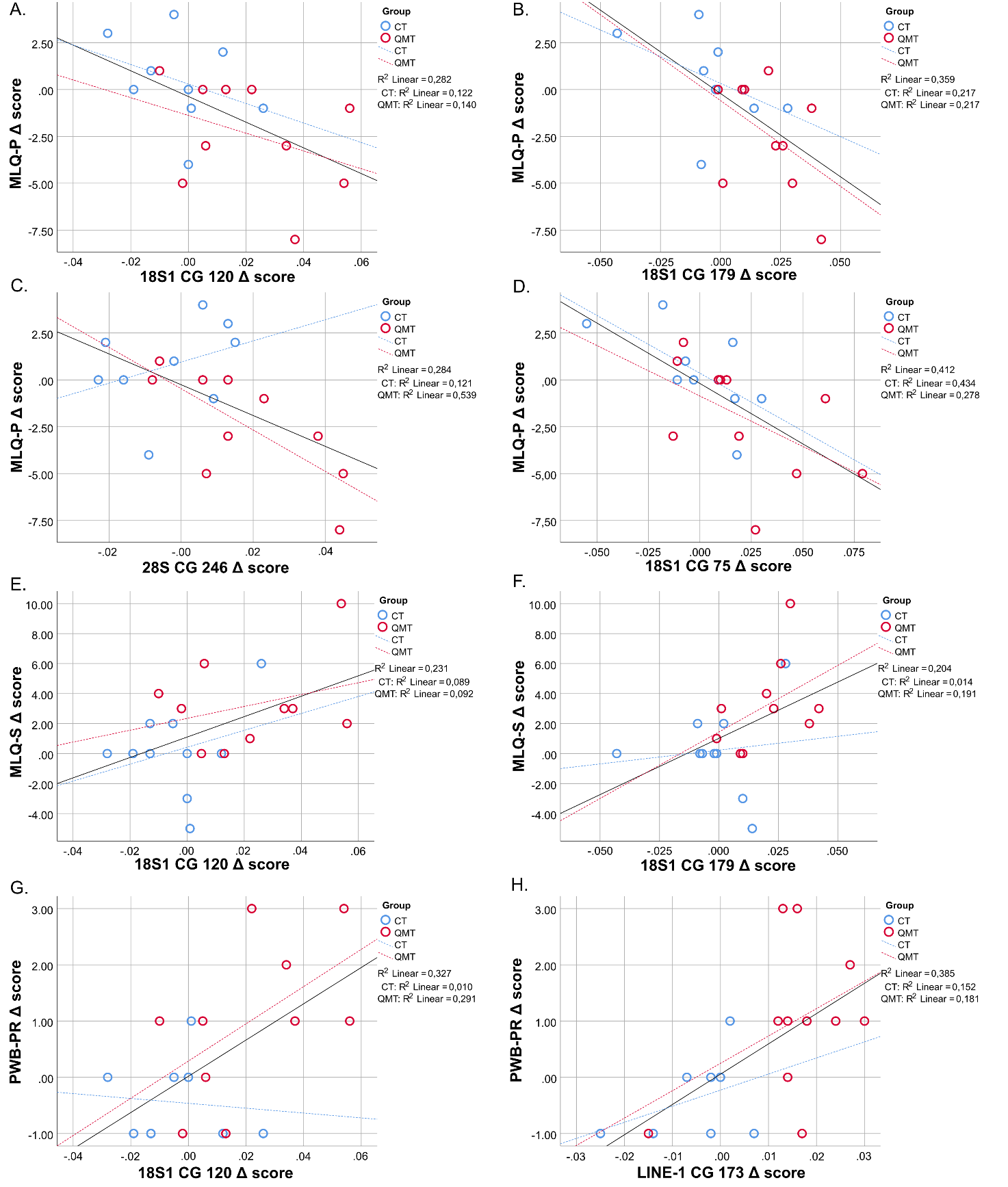


**Fig 5.** **Scatterplot representing correlations between changes in psychometric indices that showed significant Time x Group interactions in rmANCOVAs and changes in methylation levels at different sites.**

The continuous black line represents the regression line regardless of the group, while dashed colored lines represent regression for the two groups separately.

| **Group** | **Participant** | **Age** | **Sport** | **Meditation** | **Menopause** | **Smoking** | **Alcohol consumption** |
| --- | --- | --- | --- | --- | --- | --- | --- |
| QMT | Q1 | 53 | / | / | / | / | / |
|  | Q2 | 44 | / | / | / | / | / |
|  | Q6 | 51 | 4 dd/week; 6-7 hours total; >30 years | 2 dd/week; 30 minutes/session; 6 years | / | / | Daily |
|  | Q7 | 50 | 3 dd/week; 1.5 hours total; 5 years | / | Yes | / | / |
|  | Q8 | 54 | 4 dd/week; 6 hours total; 10 years | / | / | Yes | > 2 times/week |
|  | Q9 | 43 | 2 dd/week; 2 hours total; 5 years | / | / | / | / |
|  | Q11 | 54 | 2-3 dd/week; 1.5 hours total; >30 years | / | / | / | < 1 week |
|  | Q12 | 42 | 3 dd/week; 3 hours total; >30 years | / | / | Yes | 2/week |
|  | Q13 | 51 | 2 dd/week; 2 hours total; 1 year | / | / | / | / |
|  | Q15 | 47 | 1-2 dd/week; 1.5 hours total; 10 years | / | / | / | 1/week |
|  | Q16 | 47 | 1 dd/week; 1 hours total; 4 years | / | / | / | / |
| Control | C1 | 47 | 1 dd/week; 2 hours total; >20 years | / | / | / | 2/week |
|  | C2 | 52 | 3-4 dd/week; 4.5 hours total; 2 months | / | / | / | / |
|  | C3 | 52 | 2 dd/week; 2 hours total; 6 years | / | / | / | / |
|  | C6 | 49 | / | / | / | / | Daily |
|  | C7 | 40 | / | / | / | / | 2-3/months |
|  | C8 | 25 | 1-2 dd/week; 1.5 hours total; 8 years | 1 dd/week; 10 minutes/session; 8 years | / | Yes | 1/week |
|  | C9 | 55 | 2 dd/week; 2 hours total; 4 years | / | Yes | / | / |
|  | C10 | 46 | / | / | / | / | > 2 times/week |
|  | C11 | 44 | 6 dd/week; 12 hours total; 3 years | / | / | Yes | 1/week |
|  | C13 | 43 | / | / | / | / | / |
|  | C15 | 25 | 2 dd/week; 3 gours total; 2 months | / | / | / | 1/week |
|  | C16 | 26 | 3 dd/week; 6 hours total; 1 year | / | / | Yes | 2/week |
|  | C17 | 25 | 3 dd/week; 6 hours total; 1 year | / | / | Yes | 2/week |

**Table 1. Characteristics of the participants**.

| **Target ID** | **Forward Primer Sequence** | **Reverse Primer Sequence** | **n° CG Analyzed** | **Amplicon Length** | **Genome Assembly** | **Amplicon Sequence** |
| --- | --- | --- | --- | --- | --- | --- |
| *RiboProm1* | GTTTTYGTTGTGAGTTAGGTAGAGTTT | AAAAAAACRTCCCCAACCTCC | 37 | 269 | GRCh38/hg38 | GTCCCCGCTGTGAGCTAGGCAGAGCTCCGGAAAGCCCGCGGTCGTCAGCCCGGCTG GCCCGGTGGCGCCAGAGCTGTGGCGCGTCGCTTGTGAGTCACAGCTCTGGCGTGCA GGTTTATGTGGGGGAGAGGCTGTCGCTGCGCTTCTGGGCCCGCGGCGGGCGTGGG |
|  |  |  |  |  |  | GCTGCCCGGGCCGGTCGACCAGCGCGCCGTAGCTCCCGAGGCCCGAGCCGCGACCC GCGGGGACCCGCCGCGCGTGGCGCGGGAGGCTGGGGACGCCCTTCC |
| *RiboProm2* | GTGTTTTTGGGTTGATTAGAGG | CATCCCAAAACCCAACCTCTCC | 26 | 240 | GRCh38/hg38 | GTGTCCTTGGGTTGACCAGAGGGCCCCGGGCGCTCCGTGTGTGGCTGCGATGGTGG CGTTTTTGGGGACAGGTGTCCGTGTCGCGCGTCGCCTGGGCCGGCGGCGTGGTCGG TGACGCGACCTCCCGGCCCCGGGGGAGGTATATCTTTCGCTCCGAGTCGGCATTTTG |
|  |  |  |  |  |  | GGCCGCCGGGTTATTGCTGACACGCTGTCCTCTGGCGACCTGTCGCTGGAGAGGTTG GGCCTTGGGATG |
| *18S1* | TAGTYGTTTTTTAGGTTTTT | CRTACATTTATCAAATCAAA | 27 | 244 | GRCh38/hg38 | TAGCCGTTTCTCAGGCTCCCTCTCCGGAATCGAACCCTGATTCCCCGTCACCCGTGGT CACCATGGTAGGCACGGCGACTACCATCGAAAGTTGATAGGGCAGACGTTCGAATG GGTCGTCGCCGCCACGGGGGGCGTGCGATCGGCCCGAGGTTATCTAGAGTCACCAA |
|  |  |  |  |  |  | AGCCGCCGGCGCCCGCCCCCCGGCCGGGGCCGGAGAGGGGCTGACCGGGTTGGTT TTGATCTGATAAATGCACG |
| *18S2* | TGATYGGGTTGGTTTTGATTTG | TTACCTACCTAATTAATCCTACCAA | 13 | 249 | GRCh38/hg38 | TGACCGGGTTGGTTTTGATCTGATAAATGCACGCATCCCCCCCGCGAAGGGGGTCAG CGCCCGTCGGCATGTATTAGCTCTAGAATTACCACAGTTATCCAAGTAGGAGAGGAG CGAGCGACCAAAGGAACCATAACTGATTTAATGAGCCATTCGCAGTTTCACTGTACC |
|  |  |  |  |  |  | GGCCGTGCGTACTTAGACATGCATGGCTTAATCTTTGAGACAAGCATATGCTACTGG CAGGATCAACCAGGTAGGTAA |
| *28S* | GTATTTAGTTTTAGATGGAGTTTATT | AACTAACCAAAATTCCCTCA | 30 | 275 | GRCh38/hg38 | GTATTTAGCCTTAGATGGAGTTTACCACCCGCTTTGGGCTGCATTCCCAAGCAACCCG ACTCCGGGAAGACCCGGGCCCGGCGCGCCGGGGGCCGCTACCGGCCTCACACCGTC CACGGGCTGGGCCTCGATCAGAAGGACTTGGGCCCCCCACGAGCGGCGCCGGGGA |
|  |  |  |  |  |  | GCGGGTCTTCCGTACGCCACATGTCCCGCGCCCCGCCGCGGGGCGGGGATTCGGCG CTGGGCTCTTCCCTGTTCACTCGCCGTTACTGAGGGAATCCTGGTTAGTT |
| *Alu* | TTTTTATTAAAAATATAAAAATTAGT | CCAAACTAAAATACAATAA | 12 | 147 | GRCh38/hg38 | TCTCTACTAAAAATACAAAAATTAGCCGGGCGTGGTGGCGCGCGCCTGTAATCCCAG CTACTCGGGAGGCTGAGGCAGGAGAATCGCTTGAACCCGGGAGGCGGAGGTTGCA |
|  |  |  |  |  |  | GTGAGCCGAGATCGCGCCACTGCACTCCAGCCTGG |
| *LINE-1* | TTTTGAGTTAGGTGTGGGATA | CTCACTAAAAAATACCAAACAA | 18 | 245 | GRCh38/hg38 | CTCTGAGCCAGGTGTGGGATATAGTCTCGTGGTGCGCCGTTTCTTAAGCCGGTCTGA AAAGCGCAATATTCGGGTGGGAGTGACCCGATTTTCCAGGTGCGTCCGTCACCCCTT TCTTTGACTCGGAAAGGGAACTCCCTGACCCCTTGCGCTTCCCAGGTGAGGCAATGC |
|  |  |  |  |  |  | CTCGCCCTGCTTCGGCTCGCGCACGGTGCGCACACACACTGGCCTGCGCCCACTGTC TGGCACTCCCTAGTGAG |

**Table 2. Primers used in deep sequencing assay and Fasta sequence of amplicons**.

# Target Region

**Methylation levels: Mean (s.d.)** **rmANCOVA: exact p-values**

# Control QMT Main effects Interactions

**CG** **Pre** **Post** **Delta** **Pre** **Post** **Delta** **Time** **Group** **Age** **Time x Age** **Time x**

# Group

**5** 0.33 (0.01) 0.34 (0.01) 0.006 0.34 (0.01) 0.34 (0) 0.005 0.455 0.186 0.375 0.644 0.962

**27** 0.23 (0.08) 0.24 (0.09) 0.003 0.24 (0.08) 0.22 (0.06) -0.016 0.812 0.866 0.284 0.874 0.886

**36** 0.13 (0.05) 0.14 (0.06) 0.003 0.12 (0.04) 0.13 (0.05) 0.012 0.446 0.963 0.451 0.517 0.609

**38** 0.17 (0.07) 0.18 (0.08) 0.001 0.18 (0.08) 0.17 (0.07) -0.003 0.833 0.593 0.309 0.741 0.876

**42** 0.13 (0.06) 0.13 (0.06) 0.003 0.12 (0.05) 0.12 (0.04) -0.002 0.541 0.921 0.426 0.511 0.777

**50** 0.24 (0.09) 0.25 (0.09) 0.008 0.24 (0.1) 0.22 (0.06) -0.021 0.59 0.845 0.092 0.557 0.928

**59** 0.2 (0.08) 0.2 (0.08) 0.005 0.2 (0.08) 0.2 (0.07) -0.002 0.63 0.552 0.241 0.613 0.924

**65** 0.12 (0.05) 0.12 (0.05) 0 0.11 (0.04) 0.1 (0.03) -0.003 0.097 0.989 0.378 0.069 0.612

**79** 0.15 (0.05) 0.15 (0.06) 0.003 0.14 (0.04) 0.14 (0.04) 0.006 0.104 0.792 0.578 0.095 0.511

**81** 0.19 (0.07) 0.19 (0.08) 0.002 0.19 (0.07) 0.18 (0.06) -0.004 0.512 0.593 0.244 0.424 0.973

**84** 0.19 (0.06) 0.19 (0.07) 0.006 0.19 (0.05) 0.19 (0.05) 0.005 0.212 0.672 0.399 0.285 0.467

**106** 0.17 (0.06) 0.18 (0.06) 0.005 0.16 (0.05) 0.18 (0.05) 0.013 0.676 0.93 0.408 0.853 0.654

**135** 0.23 (0.07) 0.24 (0.08) 0.008 0.22 (0.05) 0.24 (0.06) 0.014 0.839 0.937 0.619 0.98 0.87

**140** 0.29 (0.09) 0.29 (0.09) 0.006 0.29 (0.07) 0.29 (0.07) 0.004 0.556 0.548 0.281 0.633 0.654

**152** 0.25 (0.08) 0.26 (0.09) 0.003 0.26 (0.08) 0.26 (0.07) 0 0.454 0.532 0.249 0.461 0.829

**154** 0.24 (0.08) 0.25 (0.09) 0.005 0.24 (0.08) 0.24 (0.07) -0.002 0.916 0.526 0.189 0.898 0.812

**157** 0.19 (0.05) 0.19 (0.06) 0.004 0.18 (0.04) 0.19 (0.05) 0.009 0.394 0.945 0.454 0.431 0.969

**RiboProm1**

**161** 0.24 (0.08) 0.24 (0.08) 0.005 0.24 (0.08) 0.24 (0.08) -0.003 0.287 0.674 0.374 0.282 0.72

**173** 0.3 (0.09) 0.3 (0.09) -0.002 0.3 (0.09) 0.28 (0.06) -0.019 0.963 0.75 0.189 0.917 0.788

**178** 0.31 (0.08) 0.31 (0.09) -0.003 0.31 (0.07) 0.31 (0.06) -0.007 0.965 0.795 0.576 0.745 0.916

**182** 0.2 (0.07) 0.2 (0.07) 0.001 0.19 (0.04) 0.2 (0.05) 0.009 0.889 0.89 0.636 0.845 0.976

**189** 0.15 (0.05) 0.15 (0.05) -0.001 0.14 (0.04) 0.14 (0.04) 0.004 0.426 0.964 0.657 0.454 0.136

**191** 0.17 (0.05) 0.17 (0.06) 0.001 0.16 (0.04) 0.16 (0.03) 0.005 0.469 0.856 0.807 0.538 0.295

**194** 0.44 (0.11) 0.44 (0.11) -0.002 0.42 (0.07) 0.41 (0.06) -0.01 0.979 0.858 0.166 0.726 0.923

**203** 0.27 (0.09) 0.28 (0.09) 0.005 0.27 (0.09) 0.26 (0.06) -0.015 0.833 0.712 0.141 0.858 0.969

**210** 0.32 (0.06) 0.34 (0.06) 0.014 0.32 (0.09) 0.27 (0.02) -0.049 0.323 0.892 **0.032** 0.598 **0.017**

**215** 0.26 (0.09) 0.26 (0.09) 0.004 0.26 (0.09) 0.26 (0.08) 0 0.775 0.459 0.172 0.786 0.906

**217** 0.19 (0.07) 0.2 (0.07) 0.003 0.2 (0.06) 0.19 (0.05) -0.005 0.51 0.749 0.429 0.457 0.636

**222** 0.22 (0.07) 0.22 (0.07) 0.001 0.21 (0.05) 0.22 (0.06) 0.011 0.537 0.96 0.415 0.54 0.541

**224** 0.26 (0.09) 0.26 (0.08) -0.003 0.26 (0.07) 0.26 (0.07) -0.004 0.743 0.526 0.264 0.956 0.798

**232** 0.28 (0.07) 0.28 (0.08) 0.001 0.28 (0.06) 0.29 (0.06) 0.001 0.862 0.603 0.376 0.841 0.703

**235** 0.37 (0.1) 0.37 (0.1) 0.002 0.37 (0.09) 0.34 (0.06) -0.024 0.583 0.856 0.205 0.439 0.952

**237** 0.3 (0.08) 0.3 (0.09) 0.003 0.3 (0.08) 0.29 (0.05) -0.014 0.298 0.877 0.299 0.295 0.519

**239** 0.38 (0.1) 0.38 (0.1) 0 0.38 (0.08) 0.38 (0.08) -0.003 0.956 0.523 0.23 0.821 0.787

**244** 0.22 (0.08) 0.22 (0.08) 0.002 0.2 (0.06) 0.22 (0.06) 0.013 0.596 0.956 0.419 0.576 0.476

**246** 0.27 (0.09) 0.27 (0.09) 0 0.28 (0.09) 0.27 (0.08) -0.007 0.935 0.484 0.179 0.696 0.767

**260** 0.65 (0.01) 0.66 (0.01) 0.003 0.66 (0.01) 0.65 (0.01) -0.004 0.692 0.75 0.412 0.688 0.284

**26** 0.34 (0.09) 0.34 (0.09) 0.002 0.34 (0.09) 0.34 (0.09) -0.002 0.182 0.631 0.298 0.177 0.242

**30** 0.19 (0.07) 0.19 (0.07) -0.001 0.18 (0.06) 0.18 (0.05) 0.004 0.536 0.969 0.557 0.569 0.392

**35** 0.19 (0.07) 0.2 (0.06) 0.007 0.18 (0.04) 0.19 (0.05) 0.014 0.448 0.783 0.858 0.655 0.929

**47** 0.32 (0.09) 0.32 (0.09) 0.002 0.32 (0.09) 0.32 (0.09) -0.002 0.202 0.683 0.372 0.198 0.332

**56** 0.12 (0.04) 0.12 (0.04) -0.001 0.14 (0.04) 0.13 (0.04) -0.007 0.2 0.286 0.445 0.239 **0.037**

**76** 0.34 (0.08) 0.34 (0.08) 0.001 0.34 (0.07) 0.35 (0.07) 0.007 0.241 0.796 0.78 0.164 0.946

**81** 0.32 (0.09) 0.33 (0.09) 0.005 0.33 (0.07) 0.33 (0.07) -0.001 0.142 0.853 0.71 0.114 0.162

**83** 0.28 (0.07) 0.28 (0.08) 0.001 0.28 (0.07) 0.28 (0.06) 0.001 0.343 0.77 0.689 0.302 0.658

**85** 0.53 (0.07) 0.51 (0.08) -0.017 0.51 (0.07) 0.51 (0.06) -0.003 0.147 0.873 0.575 0.203 0.689

**88** 0.23 (0.06) 0.23 (0.06) 0 0.33 (0.07) 0.33 (0.07) 0.004 0.742 **0.004** 0.626 0.564 0.983

**97** 0.39 (0.09) 0.39 (0.09) -0.003 0.4 (0.08) 0.39 (0.07) -0.003 0.465 0.725 0.499 0.544 0.86

**100** 0.36 (0.08) 0.36 (0.08) -0.001 0.36 (0.06) 0.36 (0.07) 0.003 0.445 0.756 0.649 0.418 0.99

**RiboProm2**

**103** 0.4 (0.09) 0.4 (0.1) -0.002 0.4 (0.08) 0.4 (0.08) 0.001 0.995 0.595 0.315 0.964 0.677

**109** 0.4 (0.08) 0.4 (0.09) 0.002 0.4 (0.07) 0.4 (0.06) 0.001 0.772 0.782 0.565 0.827 0.982

**115** 0.33 (0.09) 0.34 (0.09) 0.005 0.34 (0.08) 0.34 (0.07) 0.002 0.846 0.749 0.512 0.73 0.661

**117** 0.29 (0.08) 0.29 (0.08) 0.002 0.29 (0.07) 0.29 (0.07) 0.006 0.751 0.711 0.543 0.877 0.589

**125** 0.35 (0.1) 0.35 (0.1) 0.002 0.35 (0.09) 0.35 (0.09) -0.001 0.779 0.591 0.245 0.752 0.579

**131** 0.35 (0.1) 0.35 (0.1) 0.001 0.35 (0.09) 0.34 (0.09) -0.009 0.857 0.756 0.409 0.946 0.493

**149** 0.32 (0.09) 0.32 (0.1) 0.001 0.32 (0.09) 0.33 (0.09) 0.01 0.784 0.626 0.456 0.636 0.601

**154** 0.43 (0.1) 0.43 (0.11) 0.004 0.43 (0.09) 0.44 (0.09) 0.005 0.562 0.642 0.344 0.719 0.752

**159** 0.39 (0.11) 0.39 (0.1) -0.002 0.39 (0.09) 0.39 (0.1) -0.004 0.914 0.632 0.305 0.952 0.868

**172** 0.19 (0.07) 0.19 (0.07) -0.002 0.2 (0.07) 0.19 (0.05) -0.002 0.575 0.905 0.575 0.75 0.096

**175** 0.35 (0.1) 0.35 (0.1) -0.002 0.35 (0.11) 0.36 (0.1) 0.006 0.828 0.598 0.393 0.868 0.478

**191** 0.23 (0.08) 0.23 (0.08) 0.001 0.22 (0.05) 0.21 (0.05) -0.003 0.598 0.863 0.594 0.562 0.791

**204** 0.23 (0.08) 0.23 (0.09) -0.006 0.21 (0.08) 0.16 (0.04) -0.044 0.155 0.271 0.634 0.35 **0.029**

**212** 0.26 (0.08) 0.27 (0.08) 0.001 0.27 (0.07) 0.28 (0.07) 0.012 0.948 0.661 0.633 0.775 0.489

**4** 0.34 (0.01) 0.34 (0.01) 0.001 0.34 (0.01) 0.34 (0.01) 0.006 0.901 0.889 0.57 0.714 0.463

**24** 0.43 (0.11) 0.42 (0.11) -0.008 0.43 (0.13) 0.44 (0.12) 0.006 0.519 0.794 0.771 0.563 0.421

**30** 0.45 (0.12) 0.44 (0.11) -0.01 0.45 (0.14) 0.45 (0.13) 0 0.817 0.739 0.603 0.861 0.848

**45** 0.39 (0.11) 0.38 (0.11) -0.01 0.4 (0.14) 0.4 (0.13) 0.004 0.937 0.781 0.73 0.965 0.669

**52** 0.3 (0.1) 0.3 (0.1) -0.003 0.28 (0.08) 0.29 (0.09) 0.016 0.291 0.616 0.872 0.459 0.151

**72** 0.53 (0.11) 0.52 (0.11) -0.01 0.53 (0.13) 0.55 (0.12) 0.014 0.7 0.67 0.582 0.793 0.276

**75** 0.39 (0.1) 0.39 (0.1) -0.007 0.4 (0.12) 0.42 (0.1) 0.021 0.209 0.71 0.814 0.327 **0.036**

**85** 0.52 (0.11) 0.51 (0.11) -0.008 0.52 (0.13) 0.53 (0.13) 0.012 0.602 0.685 0.606 0.679 0.25

**104** 0.32 (0.1) 0.31 (0.09) -0.005 0.3 (0.09) 0.31 (0.09) 0.017 0.464 0.65 0.799 0.616 0.167

**108** 0.45 (0.11) 0.46 (0.09) 0.011 0.45 (0.14) 0.47 (0.12) 0.023 0.36 0.971 0.776 0.484 0.089

**117** 0.27 (0.08) 0.26 (0.08) -0.005 0.24 (0.09) 0.25 (0.08) 0.007 0.206 0.545 0.819 0.232 0.3

**120** 0.31 (0.09) 0.3 (0.08) -0.006 0.28 (0.09) 0.3 (0.08) 0.022 0.467 0.665 0.785 0.749 **0.016**

**123** 0.36 (0.1) 0.36 (0.1) -0.003 0.33 (0.1) 0.35 (0.09) 0.017 0.214 0.71 0.936 0.327 0.122

**18S1**

**128** 0.33 (0.1) 0.34 (0.08) 0.009 0.3 (0.1) 0.32 (0.09) 0.017 0.339 0.419 0.903 0.493 0.081

**135** 0.34 (0.11) 0.34 (0.1) -0.003 0.32 (0.11) 0.36 (0.13) 0.04 0.211 0.649 0.955 0.335 0.206

**139** 0.43 (0.11) 0.44 (0.09) 0.011 0.43 (0.15) 0.45 (0.13) 0.015 0.593 0.99 0.76 0.755 0.262

**143** 0.4 (0.11) 0.4 (0.11) -0.001 0.38 (0.11) 0.41 (0.13) 0.033 0.597 0.721 0.933 0.741 0.651

**148** 0.3 (0.1) 0.29 (0.09) -0.006 0.27 (0.09) 0.27 (0.1) 0 0.943 0.532 0.939 0.939 0.973

**173** 0.33 (0.1) 0.32 (0.1) -0.007 0.31 (0.09) 0.33 (0.09) 0.023 0.706 0.828 0.949 0.952 0.082

**176** 0.45 (0.12) 0.44 (0.11) -0.005 0.43 (0.1) 0.47 (0.12) 0.045 0.512 0.846 0.951 0.718 0.128

**179** 0.23 (0.07) 0.23 (0.07) -0.002 0.21 (0.06) 0.23 (0.06) 0.02 0.153 0.771 0.889 0.347 **0.007^a^**

**183** 0.31 (0.1) 0.31 (0.1) -0.007 0.29 (0.09) 0.31 (0.09) 0.016 0.365 0.699 0.905 0.462 0.171

**190** 0.53 (0.12) 0.54 (0.1) 0.009 0.53 (0.14) 0.54 (0.14) 0.013 0.65 0.937 0.586 0.738 0.323

**194** 0.46 (0.12) 0.46 (0.12) -0.004 0.47 (0.14) 0.48 (0.14) 0.013 0.81 0.693 0.586 0.958 0.497

**200** 0.47 (0.12) 0.48 (0.1) 0.011 0.47 (0.15) 0.49 (0.14) 0.024 0.508 0.926 0.684 0.66 0.15

**215** 0.43 (0.11) 0.44 (0.09) 0.013 0.43 (0.14) 0.45 (0.13) 0.017 0.372 0.966 0.684 0.525 0.204

**242** 1 (0) 1 (0) 0 1 (0) 1 (0) 0 0 0 0 0 0

**4** 0.38 (0.01) 0.39 (0.01) 0.007 0.38 (0.01) 0.38 (0.01) 0.002 0.48 0.073 0.818 0.338 0.147

**31** 0.14 (0.07) 0.14 (0.07) 0 0.14 (0.06) 0.14 (0.07) 0 0.535 0.641 0.356 0.54 0.926

**42** 0.1 (0.06) 0.11 (0.05) 0.01 0.1 (0.04) 0.1 (0.05) 0.003 **0.03** 0.969 0.757 **0.046** 0.751

**44** 0.22 (0.09) 0.23 (0.09) 0.001 0.23 (0.08) 0.23 (0.08) 0.003 0.412 0.6 0.365 0.379 0.673

**57** 0.08 (0.05) 0.09 (0.04) 0.006 0.14 (0.06) 0.13 (0.05) -0.008 0.056 **0.049** 0.862 0.078 0.578

**61** 0.21 (0.09) 0.21 (0.09) 0 0.21 (0.09) 0.22 (0.09) 0.004 0.779 0.544 0.308 0.751 0.344

**18S2**

**64** 0.15 (0.08) 0.16 (0.07) 0.011 0.24 (0.09) 0.24 (0.08) 0.001 0.102 **0.026** 0.684 0.127 0.889

**114** 0.26 (0.1) 0.26 (0.1) 0.001 0.26 (0.09) 0.26 (0.09) -0.005 0.923 0.633 0.292 0.965 0.662

**118** 0.31 (0.12) 0.3 (0.12) -0.001 0.23 (0.07) 0.23 (0.07) -0.003 0.558 0.255 0.373 0.566 0.44

**154** 0.3 (0.1) 0.3 (0.1) 0 0.3 (0.08) 0.3 (0.08) 0.005 0.634 0.665 0.436 0.669 0.19

**170** 0.37 (0.11) 0.37 (0.11) 0.001 0.37 (0.1) 0.37 (0.1) -0.006 0.388 0.711 0.374 0.288 0.928

**174** 0.35 (0.11) 0.35 (0.11) 0 0.35 (0.11) 0.36 (0.1) 0.006 0.103 0.616 0.367 0.112 0.113

**178** 0.41 (0.12) 0.41 (0.11) 0 0.41 (0.09) 0.4 (0.09) -0.007 0.874 0.744 0.416 0.716 0.661

**29** 0.16 (0.06) 0.14 (0.05) -0.018 0.15 (0.04) 0.17 (0.05) 0.017 0.919 0.7 0.928 0.815 0.331

**56** 0.27 (0.1) 0.25 (0.09) -0.017 0.27 (0.09) 0.28 (0.09) 0.005 0.48 0.532 0.672 0.48 0.121

**62** 0.31 (0.11) 0.29 (0.09) -0.018 0.31 (0.1) 0.32 (0.1) 0.006 0.669 0.518 0.648 0.668 0.172

**72** 0.26 (0.1) 0.24 (0.08) -0.015 0.26 (0.09) 0.26 (0.1) 0.003 0.883 0.594 0.684 0.858 0.413

**78** 0.31 (0.1) 0.3 (0.09) -0.016 0.31 (0.1) 0.32 (0.1) 0.008 0.911 0.554 0.669 0.95 0.099

**81** 0.13 (0.05) 0.12 (0.05) -0.004 0.11 (0.05) 0.13 (0.04) 0.016 0.784 0.889 0.813 0.591 0.297

**83** 0.14 (0.06) 0.13 (0.06) -0.011 0.12 (0.05) 0.13 (0.05) 0.002 0.553 0.856 0.946 0.496 0.214

**86** 0.43 (0.12) 0.44 (0.09) 0.005 0.43 (0.1) 0.44 (0.1) 0.005 0.763 0.884 0.583 0.792 0.477

**93** 0.2 (0.08) 0.19 (0.08) -0.015 0.19 (0.07) 0.2 (0.06) 0.012 0.465 0.984 0.957 0.521 0.078

**99** 0.19 (0.08) 0.17 (0.07) -0.012 0.17 (0.06) 0.18 (0.05) 0.006 0.919 0.843 0.761 0.95 0.188

**110** 0.18 (0.07) 0.17 (0.07) -0.012 0.17 (0.06) 0.19 (0.07) 0.019 0.413 0.9 0.8 0.409 0.572

**116** 0.25 (0.1) 0.24 (0.09) -0.016 0.25 (0.11) 0.26 (0.11) 0.005 0.535 0.565 0.664 0.538 0.259

**128** 0.25 (0.1) 0.24 (0.09) -0.014 0.26 (0.1) 0.26 (0.1) 0.004 0.97 0.619 0.786 0.972 0.288

**153** 0.27 (0.1) 0.26 (0.09) -0.014 0.27 (0.1) 0.27 (0.11) -0.007 0.59 0.703 0.714 0.738 0.64

**157** 0.26 (0.1) 0.25 (0.09) -0.01 0.26 (0.09) 0.24 (0.07) -0.023 0.808 0.849 0.946 0.727 0.638

**28S**

**160** 0.12 (0.05) 0.11 (0.05) -0.007 0.11 (0.04) 0.11 (0.04) 0.001 0.889 0.705 0.745 0.938 0.538

**163** 0.37 (0.12) 0.36 (0.1) -0.006 0.37 (0.11) 0.37 (0.1) -0.001 0.94 0.721 0.692 0.975 0.465

**170** 0.17 (0.07) 0.16 (0.07) -0.009 0.15 (0.06) 0.16 (0.06) 0.006 0.582 0.836 0.951 0.7 0.268

**179** 0.4 (0.12) 0.39 (0.1) -0.01 0.4 (0.1) 0.4 (0.1) 0.005 0.572 0.764 0.937 0.626 0.577

**183** 0.29 (0.08) 0.3 (0.06) 0.007 0.46 (0.1) 0.45 (0.1) -0.004 0.914 **0.001** 0.825 0.996 0.706

**195** 0.23 (0.1) 0.22 (0.09) -0.011 0.24 (0.11) 0.22 (0.08) -0.019 0.555 0.888 0.97 0.57 0.624

**197** 0.28 (0.1) 0.26 (0.09) -0.017 0.28 (0.1) 0.28 (0.1) -0.002 0.795 0.698 0.888 0.691 0.627

**202** 0.26 (0.09) 0.24 (0.08) -0.012 0.24 (0.06) 0.26 (0.08) 0.016 0.944 0.831 0.828 0.89 0.658

**205** 0.35 (0.11) 0.33 (0.1) -0.012 0.35 (0.11) 0.36 (0.11) 0.009 0.588 0.599 0.715 0.683 0.293

**207** 0.26 (0.09) 0.25 (0.08) -0.009 0.26 (0.1) 0.24 (0.07) -0.015 0.88 0.803 0.857 0.959 0.697

**212** 0.28 (0.1) 0.27 (0.08) -0.008 0.26 (0.07) 0.29 (0.09) 0.027 0.853 0.867 0.812 0.719 0.539

**220** 0.37 (0.12) 0.35 (0.11) -0.013 0.37 (0.11) 0.37 (0.11) 0.007 0.886 0.607 0.672 0.974 0.491

**223** 0.16 (0.06) 0.15 (0.06) -0.002 0.15 (0.04) 0.17 (0.05) 0.023 0.222 0.989 0.945 0.423 0.065

**246** 0.19 (0.07) 0.18 (0.07) -0.011 0.17 (0.06) 0.19 (0.06) 0.018 0.309 0.953 0.945 0.467 **0.015**

**249** 0.38 (0.11) 0.36 (0.11) -0.013 0.38 (0.1) 0.38 (0.1) 0.006 0.813 0.628 0.689 0.878 0.553

**26** 0.23 (0.01) 0.24 (0.01) 0.005 0.24 (0.01) 0.24 (0.02) 0.008 **0.001** 0.388 0.702 **0.002** 0.103

**30** 0.36 (0.02) 0.36 (0.02) -0.003 0.35 (0.01) 0.35 (0.01) -0.003 0.721 0.287 0.333 0.774 0.711

**38** 0.26 (0.02) 0.25 (0.01) -0.004 0.25 (0.01) 0.26 (0.02) 0.008 0.263 0.557 0.579 0.305 0.088

**40** 0.22 (0.02) 0.22 (0.01) 0 0.22 (0.01) 0.22 (0.02) -0.003 0.1 0.381 0.742 0.067 0.309

**42** 0.27 (0.01) 0.28 (0.01) 0.013 0.27 (0.02) 0.28 (0.02) 0.006 0.378 0.667 0.966 0.664 0.479

**62** 0.28 (0.02) 0.27 (0.01) -0.009 0.28 (0.01) 0.27 (0.01) -0.009 0.116 0.532 0.907 0.252 0.448

**Alu**

**84** 0.33 (0.01) 0.33 (0.01) 0.005 0.33 (0.02) 0.33 (0.02) 0.003 0.925 0.581 0.335 0.932 0.89

**94** 0.26 (0.01) 0.28 (0.01) 0.015 0.27 (0.01) 0.27 (0.01) 0.001 0.44 0.545 0.78 0.244 **0.032**

**101** 0.31 (0.01) 0.31 (0.02) -0.001 0.31 (0.01) 0.32 (0.01) 0.005 **0.003** 0.944 0.155 **0.002** 0.345

**118** 0.37 (0.01) 0.38 (0.02) 0.003 0.37 (0.01) 0.38 (0.01) 0.005 0.778 0.501 0.235 0.897 0.759

**124** 0.37 (0.02) 0.37 (0.01) -0.004 0.37 (0.01) 0.37 (0.02) -0.007 0.553 0.748 0.957 0.708 0.704

**126** 0.31 (0.01) 0.32 (0.01) 0.01 0.3 (0.02) 0.3 (0.01) 0 0.313 0.285 0.258 0.425 0.509

**27** 0.74 (0.02) 0.74 (0.03) 0.005 0.73 (0.03) 0.73 (0.02) 0.004 0.479 0.638 0.625 0.429 0.481

**34** 0.79 (0.01) 0.79 (0.03) 0.001 0.79 (0.02) 0.8 (0.01) 0.008 0.244 0.822 0.39 0.194 0.951

**37** 0.81 (0.02) 0.8 (0.03) -0.002 0.8 (0.01) 0.8 (0.01) -0.004 0.872 0.922 0.579 0.846 0.689

**49** 0.7 (0.02) 0.69 (0.02) -0.005 0.69 (0.02) 0.7 (0.02) 0.004 0.5 0.477 0.896 0.515 0.141

**61** 0.82 (0.01) 0.82 (0.02) -0.002 0.82 (0.01) 0.82 (0.01) -0.001 0.978 0.775 0.171 0.97 0.956

**70** 0.54 (0.01) 0.5 (0.15) -0.042 0.81 (0.01) 0.82 (0.01) 0.002 0.469 **< 0.001** 0.453 0.364 0.202

**85** 0.8 (0.02) 0.8 (0.02) -0.002 0.8 (0.02) 0.81 (0.02) 0.007 0.277 0.6 0.079 0.348 0.376

**99** 0.75 (0.01) 0.75 (0.01) 0.001 0.75 (0.02) 0.77 (0.01) 0.021 0.35 0.157 0.89 0.74 **0.009^a^**

**LINE-1**

**103** 0.71 (0.01) 0.71 (0.02) 0.001 0.7 (0.01) 0.7 (0.02) 0.005 0.767 0.734 0.507 0.874 0.573

**123** 0.79 (0.01) 0.78 (0.03) -0.006 0.78 (0.02) 0.79 (0.01) 0.008 0.323 0.818 0.613 0.281 0.668

**149** 0.83 (0.02) 0.82 (0.02) -0.008 0.77 (0.01) 0.76 (0.01) -0.005 0.485 **< 0.001** 0.634 0.549 0.989

**173** 0.78 (0.01) 0.78 (0.01) -0.001 0.77 (0.01) 0.79 (0.01) 0.015 0.777 0.202 0.227 0.876 **0.004^a^**

**183** 0.79 (0.02) 0.79 (0.02) 0 0.79 (0.02) 0.79 (0.01) -0.001 0.939 0.853 0.35 0.929 0.975

**188** 0.8 (0.02) 0.81 (0.02) 0.005 0.8 (0.01) 0.8 (0.02) 0.003 0.631 0.886 0.576 0.863 0.893

**190** 0.76 (0.01) 0.76 (0.02) -0.003 0.76 (0.02) 0.76 (0.01) -0.005 0.064 0.38 0.626 0.104 0.562

**194** 0.76 (0.01) 0.76 (0.02) 0 0.77 (0.01) 0.76 (0.02) -0.005 0.806 0.701 0.778 0.893 0.626

**199** 0.79 (0.01) 0.79 (0.02) -0.002 0.79 (0.02) 0.79 (0.02) 0.006 0.931 0.761 0.339 0.937 0.572

**217** 0.83 (0.01) 0.83 (0.02) -0.004 0.83 (0.01) 0.84 (0.01) 0.007 0.418 0.331 0.502 0.496 0.212

**Table 3.** **CG methylation level for each DNA target and rmANCOVA analysis**.

Left side: mean and standard deviation of methylation values for each site at T1, T2 and delta scores (Δ = T2 – T1) in the Control and QMT groups. Right side: exact p-values related to the main effects of Age, Time, Group and interaction of Time x Group and Time x Age following the rmANCOVAs. ^a^Time x Group interactions that survived the FDR correction.

| **RiboProm1** | **RiboProm2** |  | **18S1** |  | **28S** | **Alu** | **LINE-1** |  |
| --- | --- | --- | --- | --- | --- | --- | --- | --- |
| Δ **CG 210** | Δ **CG 56** Δ **CG 204** | Δ **CG 75** | Δ **CG 120** | Δ **CG 179** | Δ **CG 246** | Δ **CG 94** | Δ **CG 99** | Δ **CG 173** |

Pearson

| r | tailed) | r | tailed) | r | tailed) | tailed) | r | tailed) |  | tailed) r | tailed) | r | tailed) | r | tailed) |  | tailed) |
| --- | --- | --- | --- | --- | --- | --- | --- | --- | --- | --- | --- | --- | --- | --- | --- | --- | --- |
| Δ **MLQ-P** 0.308  **MLQ** | 0.2 | 0.249 | 0.304 | 0.28 | 0.219 | **-0.642** 0.002^a^** | **-0.531** | **0.019** | **-0.599**** | **0.007^a^** **-0.533*** | **0.019** | 0.392 | 0.087 | -0.425 | 0.055 | -0.379 | 0.109 |
| Δ **MLQ-S** -0.262 | 0.279 | 0.04 | 0.868 | -0.325 | 0.139 | 0.419 0.059 | **0.481*** | **0.032** | **0.451*** | **0.046** 0.239 | 0.31 | -0.007 | 0.978 | 0.421 | 0.051 | 0.336 | 0.148 |
| **PWB** Δ **PWB-PR** -0.115 | 0.648 | 0.114 | 0.642 | -0.232 | 0.312 | 0.352 0.128 | **0.572*** | **0.011^a^** | 0.351 | 0.141 0.399 | 0.091 | -0.254 | 0.279 | 0.276 | 0.227 | **0.620**** | **0.005^a^** |

Sig. (2-

Pearson

Sig. (2-

Pearson

Sig. (2-

Pearson r Sig. (2-

Pearson

Sig. (2-

Pearson r Sig. (2-

Pearson

Sig. (2-

Pearson

Sig. (2-

Pearson

Sig. (2-

Pearson r Sig. (2-

**Table 4.** **Correlation between Psychometric Variables and CG Methylation Levels**. ^a^Correlation that survived the FDR correction.
